# TPS5 and TOR signaling components are determinants of *Populus balsamifera* leaf morphology

**DOI:** 10.3389/fpls.2025.1683866

**Published:** 2025-12-04

**Authors:** Marc J. Champigny, Shankar Pahari, Charles A. Hefer, Salim N. Silim, Shawn D. Mansfield, Raju Y. Soolanayakanahally

**Affiliations:** 1 PhenoLogic Co., Toronto, ON, Canada; 2 Saskatoon Research and Development Centre, Agriculture and Agri-Food Canada, Saskatoon, SK, Canada; 3 Bioeconomy Science Institute, AgResearch Ltd., Lincoln, New Zealand; 4 Ottawa Research and Development Centre, Agriculture and Agri-Food Canada, Ottawa, ON, Canada; 5 Department of Wood Science, University of British Columbia, Vancouver, BC, Canada; 6 Department of Botany, University of British Columbia, Vancouver, BC, Canada; 7 Indian Head Research Farm, Agriculture and Agri-Food Canada, Indian Head, SK, Canada

**Keywords:** GWAS, deep learning, leaf morphology, terpene synthase, natural variation, Populus

## Abstract

Variation in leaf morphology in plant species predicts long-term growth and yield. Consequently, we aim to understand the genetic basis of natural variation in poplar leaf morphology as an avenue to maximize biomass accrual. Multilocus GWAS and deep learning genomic prediction were used to investigate the genetic architecture of twelve correlated traits representing leaf size and shape in a diverse population of 313 *Populus balsamifera* L. genotypes. 94 significant associations were detected, with 70 associations unique to a single trait, and 24 were detected in association with more than one trait. We developed genomic selection models to predict leaf morphology in novel genotypes using a strategy called GWADL (Genome-Wide Association enriched Deep Learning). We detected significant SNP-trait associations in the poplar *TOR* orthologue and likely upstream activating kinases *SnRK3* and *SnAK1*. The most significant polymorphism, explaining variance in tip angle, leaf mass-per-area, and serration density, was detected in association with *TERPENE SYNTHASE5, PbTPS5*. Exogenous application of sesquiterpenes β-eudesmol and 1αH,5αH-Guaia-6-ene-4β,10β-diol in developing young poplar leaves resulted in significantly smaller mature leaves. This study provides a genetic and mathematical foundation for improving poplar performance by optimizing leaf morphology, and importantly identified a novel role for the sesquiterpene synthase *PbTPS5* in normal plant growth and development.

## Introduction

Leaves act as an interface between plants and the environment with respect to photosynthetic capacity, gas exchange, and thermal regulation. Leaf morphological features, such as their overall surface area, specific shape, and thickness are important determinants of photosynthetic capacity by relating cell size, chlorophyll, and RuBisCO contents per unit area exposed to sunlight (Zhu et al., 2010). Within species, populations, and individuals, there exists considerable variation in leaf morphology resulting from evolutionary strategies that maximize photosynthesis while minimizing water loss (Fritz et al., 2018). The crucial role played by leaf morphology in crop productivity was highlighted during the “Green Revolution” where unparalleled rice and wheat yield increases were due to breeding of upright, semi-dwarf varieties with increased tillering (Peng et al., 1999). The idea of enhancing plant productivity by manipulating leaf architecture has been extensively discussed in the literature (Mathan et al., 2016; Meyer and Purugganan, 2013; Nunes-Nesi et al., 2016) but has found little application outside of cereal breeding due to a limited understanding of the genetic basis of this essential developmental process in most species.

Morphology of mature leaves results from a complex network of developmental processes regulating meristem identity (Clark, 1997), polarity in three dimensions (proximal/distal, medial/lateral, adaxial/abaxial), cell division, elongation, and differentiation (Sluis and Hake, 2015). Many genes involved in initiation of leaf primordia and subsequent patterning and growth have been identified, primarily via mutagenesis studies in *Arabidopsis thaliana*. Known genes regulating meristem identity and initiating leaf primordia include the auxin transporter *PIN-FORMED1* (*PIN1*) and transcription factors *CUP-SHAPED COTYLEDON2, KNOX1*, and *WUSCHEL*. Genes involved in proximodistal patterning and outgrowth include *KNOTTED1* transcription factor and *LIGULELESS NARROW-REFERENCE* ser/thr kinase. Dorsoventral patterning is promoted by *PHANTASTICA, ASYMMETRIC LEAVES2*, and *KANADI*, as well as two non-coding RNAs, miRNA165 and miRNA166. *YABBY* transcription factor and *YUCCA* flavin mono-oxygenase play important roles in mediolateral patterning and outgrowth. Leaf developmental regulators identified by forward and reverse genetics approaches are thoroughly reviewed elsewhere (Dkhar and Pareek, 2014; Wang et al., 2021).

Mutagenesis studies suggest that multiple mechanisms exist to regulate the final shape and size of mature leaves, for example by either elongating cells or increasing cell division in one dimension (Tsukaya, 2006). The optimization of leaf form and function adapted to specific environmental conditions is thought to have arisen through natural selection over geological time (de Boer et al., 2012). It is therefore expected that analyses of natural genetic variation, in contrast, to forward and reverse genetics, will yield insight into ecologically adaptive mechanisms for regulating photosynthesis and growth through leaf morphology.

Genetic association studies of plant populations have identified several candidate genes whose activity explains natural variation in leaf morphology. Leaf shape and size traits are multigenic and complex, suggesting that many loci of small effect are responsible for variation in the architecture of plant leaves in natural populations (Chitwood et al., 2013; Gupta et al., 2019; Mähler et al., 2020). Leaves of grape, *Vitus vinifera* L., exhibit striking differences in leaf architecture among cultivars. Genes differentially expressed between leaves of different shapes can be identified, but the genetic architecture underlying heritable grape leaf morphological traits is not yet understood (Chitwood et al., 2014). Quantitative trait locus (QTL) mapping in *A. thaliana* identified 16 QTLs associated with leaf shape (Pérez-Pérez et al., 2002). In rice (*Oryza sativa* L.), Genome-Wide Association (GWA) analysis of 533 natural accessions identified several leucine zipper transcription factors associated with leaf size and polarity, as well as the polar auxin transporter *Narrow Leaf 1* (Qi et al., 2008; Yang et al., 2015). In maize (*Zea mays* L.), leaf angle and size were associated with genetic variation at two *LIGULELESS* loci contributing to more upright leaves (Tian et al., 2011).

The *Populus* genus is a favorable system for association mapping of leaf traits due to extensive natural genetic variation, as well as phenotypic variation for leaf size and shape (Stettler and Bradshaw, 1996). QTL analysis involving a pseudo-backcross between narrow-leafed black cottonwood *Populus trichocarpa* and wide-leafed eastern cottonwood *Populus deltoides* identified an ADP-ribosylation factor GTPase playing a role in vesicular trafficking (Drost et al., 2015). In quaking aspen, *Populus tremula* L., GWA was used to identify a subset of single nucleotide polymorphisms (SNPs) located within differentially expressed genes defining leaves of contrasting shapes. Results indicated that leaf morphology in this species is highly polygenic and characterized by many genes of small effect (Mähler et al., 2020). GWA of leaf morphological traits in a natural *P. trichocarpa* population identified loci enriched in GO terms associated with photosynthesis and cellular homeostasis (Chhetri et al., 2020).

Analyses of natural genomic structural variants among poplar species as well as artificially induced structural variants has revealed new insight into the genetic basis of leaf morphology variation. A super-pangenome analysis of 19 species in the *Populus* genus identified a 180 bp insertion/deletion associated with expression of *CUP-SHAPED COTYLEDON2* (*CUC2*) and leaf serrations (Shi et al., 2024). Bastiaanse and colleagues used a population of ~600 artificially induced indel lines (Henry et al., 2015) to analyze gene dosage-specific expression QTLs in the context of leaf morphological traits, resulting in a list of 116 candidate genes correlated with dosage effects on leaf development in poplar (Bastiaanse et al., 2021).

Several studies have demonstrated that leaf morphology in poplar species predicts long-term growth and yield traits (Harrington et al., 1997; Marron et al., 2005; Marron and Ceulemans, 2006). Consequently, we aim to understand the genetic basis of natural variation in poplar leaf shape as an avenue to maximize industrially important wood and bioenergy traits. We employed genome-wide association and deep learning genomic prediction approaches to map the genetic architecture of twelve correlated traits representing leaf size and shape in a diverse population of 313 *Populus balsamifera* L. (balsam poplar) genotypes. A multilocus GWA algorithm known to minimize both false negatives and false positives, FarmCPU (Fixed and random model Circulating Probability Unification) (Liu et al., 2016) was used to identify marker-trait associations from a panel of 5,641,729 SNPs revealed by whole genome resequencing. Significant SNP-trait associations were detected in the poplar *TOR* (*Target Of Rapamycin*) orthologue, as well as likely upstream activating kinases *SnRK3* and *SnAK1*. The most significant association, explaining variance in total leaf area, leaf thickness, and serration density, was detected in an exon of *TERPENE SYNTHASE5, PbTPS5*. Deep learning genomic prediction resulted in highly accurate estimates of leaf shape and size, and also found the *PbTPS5*-associated polymorphism to be the most important predictor of leaf morphology. Exogenous application of sesquiterpenes β-eudesmol and 1αH,5αH-Guaia-6-ene-4β,10β-diol in developing poplar leaves resulted in significantly smaller mature leaves. Our study provides a genetic and mathematical foundation for improving poplar performance through the rational optimization of leaf shape, and identified *PbTPS5* as a novel candidate regulator of leaf development.

## Materials and methods

### Growth and phenotyping of *P. balsamifera*

*P. balsamifera* used in this study comprises a subset of a larger Agriculture Canada Balsam Poplar (AgCanBaP) collection of 65 provenances (Soolanayakanahally et al., 2009). A common garden was established at Indian Head, Saskatchewan (50.52°N, 103.68°W; elevation 605 m), in May 2007. Fifteen genotypes from each provenance were planted in a group at 2m × 2m spacing, and groups (provenances) were then randomized within blocks (*i.e.*, five ramets per genotype). The current study employed a subset of 313 different genotypes sourced from AgCanBaP collection.

Towards leaf phenotyping, the third fully expanded leaf was selected. The leaves were collected in brown paper bags, placed in a cooler box filled with ice, and moved to the laboratory. Individual leaf area (mm^2^) was measured using LI-COR Li-3100C Area Meter. The leaf length (mm), leaf width (mm), laminar length (mm), petiole length (mm), petiole width (mm), widest point (mm), and length/width was recorded using a digital sliding caliper. A protractor was used to measure base angle and tip angle. The serration density (cm^-1^) was computed with the help of a magnifying glass and measuring scale. This activity was completed over a 2-week period. We calculated leaf mass area (mg/cm^-2^) by oven drying the leaves at 60 °C to constant mass.

### Genome sequencing of *P. balsamifera* DNA

For whole genome sequencing, a young leaf from growing tips was collected from the same balsam poplar genotypes in June 2011. Leaf tissue was flash-frozen in liquid N_2_, transported to the University of British Columbia, and stored at -80 °C until further use.

Frozen tissue samples were ground to a fine powder in liquid N_2_ using pre-chilled mortar and pestles. DNA was extracted from 100 mg of each powdered sample using the CTAB method (Murray and Thompson, 1980). Purified DNA samples were sequenced at the Michael Smith Genome Science Centre (Vancouver, Canada). Sequencing libraries were constructed using a TruSeq DNA HT (Illumina) sample kit, the original Illumina adaptors, and with a target insert sequence of 300 nucleotides. DNA was sequenced, 2–3 samples per lane, at a target read length of 125 cycles/end on an Illumina HiSeq2500 instrument.

Following an initial filter selecting for Illumina chastity-passed sequences, adaptors, and ambiguous bases were removed from reads with Trimmomatic v0.30 (Bolger et al., 2014) in paired-end mode using the ILLUMINACLIP algorithm with default parameters. Unpaired reads, as well as those of length < 36 nt, were excluded from further analyses.

### SNP identification

Illumina sequence data was aligned to the *P*. *trichocarpa* reference genome (V3.0, http://www.phytozome.net) assembly using BWA (version 0.6.1) (Li and Durbin, 2009) with a maximum mis-alignment threshold of 4 bp, restricting insertions or deletions within 5 bp of the read boundary, and a maximum insert size of 500 bp. Paired-end mates we re-synced using FixMateInformation by Picard-tools (http://picard.sourceforge.net/), followed by local re-alignment by GATK version 1.5 (DePristo et al., 2011). The UnifiedGenotyper from the GATK was used to identify variants with a minimum phred-scaled confidence value of 30, and all SNPs with read coverage >=5 reads were retained for downstream analysis.

### Heritability of leaf traits

Broad-sense heritability (*H^2^*) estimates of leaf morphological traits were obtained using the repeatability method (R package “heritability” v.1.3). Repeatability or intra-class correlation was estimated by *Vp = (Vg +Ve)*, where *Vg = (MS(G) - MS(E))/r*, and *Ve = MS(E)*.

Where, *r* is the number of replicates per genotype, and *MS(G)* and *MS(E)* are the mean sums of squares for genotype and residual error obtained from ANOVA. Broad-sense heritability of individual observations (*H^2indiv^*) is given by *σ^2G^/(σ^2G^ + σ^2E^*). Broad-sense heritability of genotype means across blocks (*H^2geno^* or line-level heritability) is given by *σ^2G^/(σ^2G^ + σ^2E^/r*).

### Multivariate statistical analyses

Box plots were produced using the multcompView v.0.1–7 R package. Significant differences among groups were calculated *post hoc* according to Tukey’s HSD test. Hierarchical clustering of 500,000 maf- and LD-filtered SNPs was conducted with pvclust v2.0.0 and pheatmap v1.0.8 R packages using the Ward clustering method computed on Euclidean distances. Admixture analyses were conducted on the subset of 500,000 SNPs (R package “LEA” v.2.8) with default parameters. Venn-type set diagrams illustrating overlap among subsets of SNPs were produced with the UpSetR v.1.4.0 R package.

### GWAS

From the 54,637,374 SNPs identified in at least one individual, a total of 5,641,729 bi-allelic SNPs were filter-selected, where: a) fewer than 5% SNP calls absent among the 313 genotypes, b) polymorphism is present as only a single minor allele, and c) minor allele frequency is greater than 5%. Missing data (2.8% of total) was imputed as the mean among 313 genotypes. Phenotypic data was exponentiated using the Box-Cox transformation to obtain a normalized distribution. Values of the exponent λ were tested in increments of 0.1 between -5 and +5. Normality of the resulting distributions was estimated and selected using the Shapiro-Wilk statistic. GWA was conducted on each of 12 Box-Cox transformed leaf morphological traits using 5,641,729 SNPs with minor allele frequency > 0.05. GWA used an iterative fixed and random effects model in additive mode (FarmCPU) (Liu et al., 2016). Significant associations were selected after correction for multiple testing using the stringent Bonferroni threshold, α = 0.05/5,641,729, *P* < 8.8 × 10^-9^. Significant associations were annotated by identifying the nearest gene model in the Phytozome v3.0 *P. trichocarpa* reference genome. Biological functions of poplar gene models were inferred by the most significant BLAST match in the TAIR10 *A*. *thaliana* reference genome.

### Genomic prediction by GBLUP and GWADL

Quantitative estimates of poplar leaf traits based on SNP genotyping were modelled by constructing supervised feed-forward artificial neural networks in R using H2O.ai v3.34.07 (http://h2o.ai). Models with two hidden layers were trained with an input layer consisting of SNPs selected by *P*-value enrichment from GWA, or an equivalent number of widely spaced, uncorrelated SNPs selected by choosing every *n*^th^ SNP in the dataset. Output layers consisted of a single output neuron for regression predictions. Models were trained to minimize the mean squared error (MSE) loss function using a parallelized method of stochastic gradient descent computed by backpropagation (LeCun et al., 1998). Although the maximum number of epochs used for training was 200, best-performing models were selected based on an early stopping criterion of < 0.01 MSE reduction over 3 epochs. 5-fold cross-validation performance for each model was estimated using the CV split method in scikit-learn 0.23.1. The algorithm of Gedeon (Gedeon, 1997) was used to score/rank the relative importance of SNPs in the models.

Model tuning parameters were assayed using grid searches to select among 3,840 architectures for each trait: a) the number of neurons in each hidden layer ranged from 40 to 300, b) the activation function used at each hidden layer was Tanh, rectified linear units, or maxout, c) the value of the L_1_ (Lasso) regularization parameter ranged from 2e^-6^ to 1e^-4^, d) the value of the L_2_ (Ridge) regularization parameter ranged from 2e^-6^ to 1e^-4^, and e) the proportion of neurons randomly dropped from the input layer and hidden layers with each training epoch ranged from 4% to 50%.

Genomic Best Linear Unbiased Prediction (GBLUP) was conducted using R package rrBLUP v.6.1 (Endelman, 2011) with 5-fold cross-validation as described above. The realized population relationship matrix was obtained using the formula of VanRaden (VanRaden, 2008) and variance components were estimated by REML.

### Exogenous treatments of developing leaves with sesquiterpenes

Okanese poplar genotype were pot-grown in greenhouse conditions with sufficient soil moisture and nutrients at a temperature of 25°C during the day and 20°C at night. Cool-white fluorescent lamps were used in addition to natural light to provide a 16-hour photoperiod and a minimum photosynthetic photon flux density of 400 µmol m^−2^ s^−1^ at canopy level. After 30-days of free growth, three leaves from each plant (3^rd^, 4^th^, and 5^th^ youngest leaves) were tagged and treated with sesquiterpenes using one of three application methods, namely leaf spray, stomatal infiltration, and petiole injection.

50 mM stock solutions of α, β-elemolic acid (Sigma Aldrich CAT # PHL80435-10MG), β-eudesmol (Sigma Aldrich CAT # 17790-10MG), and 1αH,5αH-Guaia-6-ene-4β,10β-diol (Medchem Express CAT # HY-N5081) were prepared by dissolving each compound individually in dimethyl sulfoxide (DMSO, Fisher Scientific). Stock solutions were diluted 1000-fold in distilled water to make 50 uM treatment solutions. A combined treatment solution was prepared by mixing equal amounts of the three individual sesquiterpene solutions. Control (mock treatment) solution consisted of DMSO diluted in distilled water (0.1% v/v). Treatment methods were initially tested using 50 uM of the herbicide 2,4-dichclorophenoxyacetic acid (2,4-D, Sigma Aldrich CAT # D7299) to demonstrate uptake in developing leaves. All treatments were carried out under a chemical fume hood with proper aeration. For the leaf spray method, 0.5% (v/v) of 10% tween20 was added to the working solution as a surfactant. The selected leaves were thoroughly sprayed on the abaxial and adaxial surfaces. For stomatal infiltration, 500 uL of solution was infiltered in multiple spots on the abaxial surface of each leaf using a 1 mL syringe (needle removed). For petiole injection, 200 uL of the solution was injected into the petiole of each leaf using a 1 mL BD Tuberculin syringe (25 G × 5/8 inch needle, BD-309626). Treated plants were maintained in the greenhouse until the leaves were fully matured. Tagged leaves were scanned after 50 days.

Leaf morphology traits (blade area, blade perimeter, blade length, blade width, and petiole width) of treated leaves were analyzed using MorphoLeaf software (Biot et al., 2016). Effects of sesquiterpene and treatment method were statistically analyzed by 2-way ANOVA and *post-hoc* Tukey HSD test in R. Differences between means were analyzed using Welch’s t-test in R.

## Results and discussion

### Heritable natural variation in *P. balsamifera* leaf morphology

The *P. balsamifera* genotypes analyzed in this study are a subset of a larger Agriculture Canada Balsam Poplar (AgCanBaP) collection (Soolanayakanahally et al., 2009). Dormant whips harvested from 313 individuals in 32 populations were established in a symmetrical block design (each genotype replicated among 3 blocks) at a common garden site at Indian Head, Saskatchewan (Soolanayakanahally et al., 2013). Provenances selected for analysis included *P. balsamifera* genotypes sampled from a very large natural range, from Matapedia, Quebec (MAT: 67.18° W) as the easternmost extreme, to Denali National Park, Alaska (149.02° W) as the westernmost extreme, and Inuvik, Northwest Territories (INU: 68.3° N) as the northernmost extreme.

**Figure 1:**
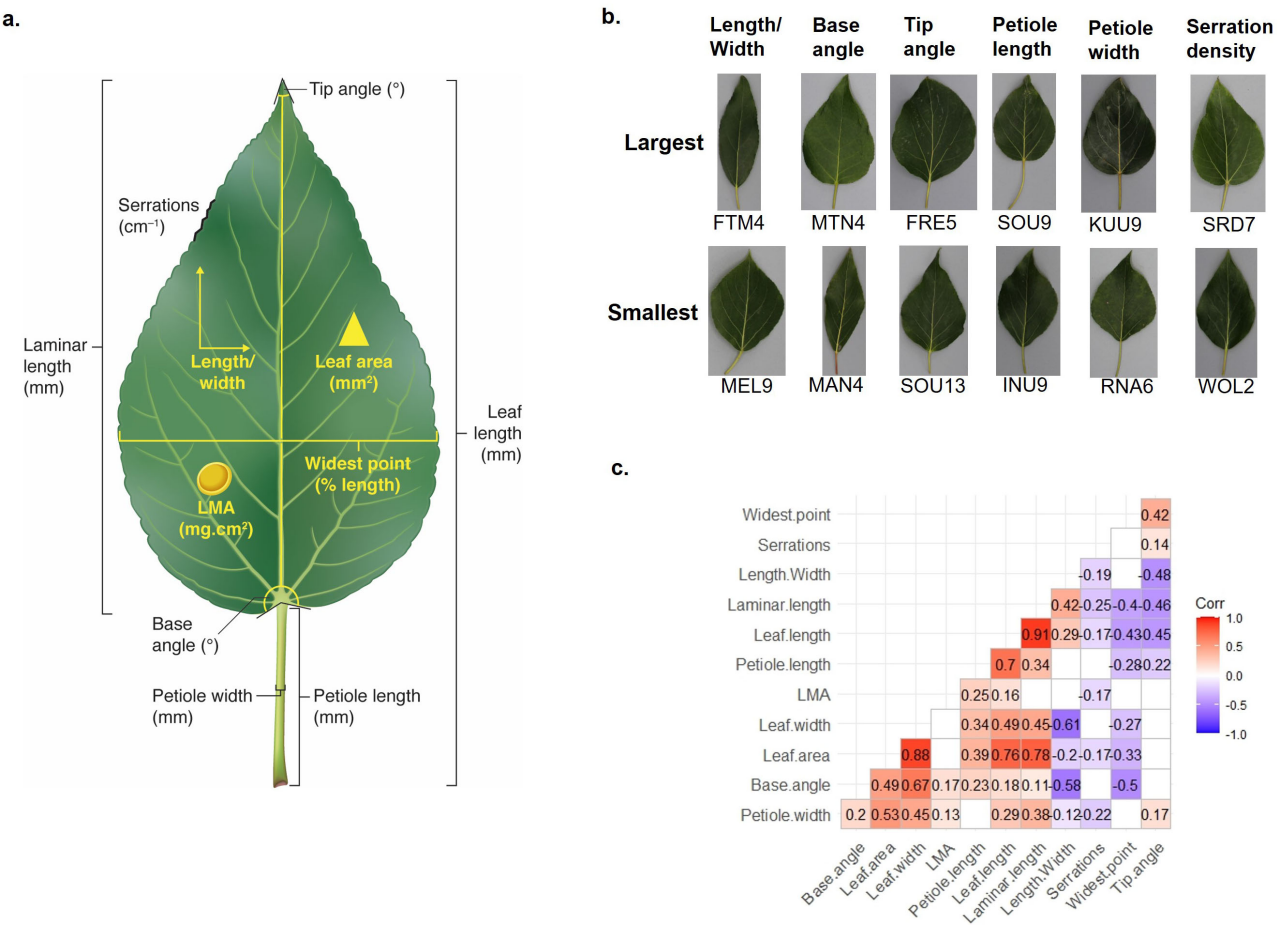
Natural variation in leaf morphology among *P. balsamifera* genotypes. **(a)** Schematic illustration of 12 leaf morphological traits measured in this study. LMA – leaf dry mass per unit area (Artwork by Debbie Maizels, Zoobotanica Scientific Illustration). **(b)** Photographs of leaves exhibiting extremes of selected leaf traits within the study population of *P. balsamifera* genotypes. **(c)** Correlations among leaf morphological traits. Significant correlations (Pearson) after correction for multiple testing are indicated.

Previous studies have highlighted the extensive natural variation in leaf morphology within and between species in the *Populus* genus (Mähler et al., 2020; Stettler and Bradshaw, 1996; Drost et al., 2015; Chhetri et al., 2020). To investigate the natural genetic basis of leaf architectural variation in the AgCanBaP collection, we evaluated leaf morphometric features using the third fully expanded leaf from a branch exposed to direct sunlight. Twelve correlated traits representing aspects of leaf size (leaf area, leaf length, leaf width, leaf mass per area, laminar length, petiole length, and petiole width) and shape (length/width, widest point, base angle, tip angle, and serration density) were measured (**Figures 1a-c**).

Multivariate statistical methods, such as principal component analysis to reduce dimensionality, have been used to represent leaf morphology as uncorrelated features (Klein and Svoboda, 2017). However, candidate genes identified by phenotypic association studies are rarely verified and the natural genetic architecture of poplar leaf morphology is not well understood. Our expectation of correlated leaf developmental traits resulting from patterns of cell division, elongation, and differentiation in three dimensions is that causative polymorphisms will explain phenotypic variance in multiple traits. We therefore examined each of the correlated leaf morphological traits using GWAS from the perspective that genuine associations will be reproducible.

Broad-sense heritabilities (*H*^2indiv^) of leaf morphological measurements across blocks ranged from 0.24 for LMA to 0.47 for leaf base angle. Our analyses found no evidence of a block effect between the largely identical blocks at the Indian Head common garden. Furthermore, there exists considerable variation in leaf morphology within individual trees (**Supplementary Figure S1**), a source of variance in leaf shape that is poorly understood in any species. We therefore studied the line-level heritabilities (*H*^2geno^), defined as the heritability of the genotype mean across blocks, to average out random effects and measurement errors. Line-level heritabilities of leaf morphological traits in *P. balsamifera* ranged from 49% for LMA, tip angle, and serrations to 73% for leaf base angle (**Table 1**).

**Table 1:** Heritability of *P. balsamifera* leaf morphological traits and correlations with geo-climactic variables.

Trait	*H^2^* indiv.	*H^2^* geno	Latitude	Longitude	Elevation	FFD	MAT	AST	MTCM	MTWM	MAP	MSP	CONT	ADI	SDI
			(°N)	(°W)	(m)	(days)	(°C)	(°C)	(°C)	(°C)	(mm)	(mm)			
Leaf area (mm^2^)	0.37	0.63	-0.11	-0.19	-0.033	0.14	0.11	-0.011	-0.069	0.16	0.12	0.12	-0.15	-0.1	-0.23
Leaf length (mm)	0.32	0.59	-0.13	-0.048	0.16	0.17	0.2	0.12	0.15	0.024	0.028	0.12	-0.16	0.043	**-0.24**
Leaf width (mm)	0.45	0.71	-0.22	**-0.31**	-0.11	0.2	0.16	0.036	0.16	0.0087	**0.28**	0.2	-0.18	-0.21	-0.049
Leaf length/width	0.45	0.71	0.2	**0.37**	**0.27**	-0.12	-0.032	0.016	-0.036	-0.041	**-0.33**	-0.21	0.024	**0.33**	0.17
Widest point (% length)	0.38	0.64	0.23	0.044	-0.21	-0.2	**-0.29**	**-0.27**	-0.19	-0.19	-0.019	-0.14	0.14	-0.066	**0.25**
LMA (mg.cm^2^)	0.24	0.49	0.22	0.15	0.03	**-0.32**	**-0.32**	-0.2	**-0.36**	-0.16	**-0.27**	-0.15	**0.34**	0.017	**0.11**
Laminar length (mm)	0.38	0.65	0.016	0.094	0.22	0.064	0.12	0.045	0.084	-0.062	-0.11	-0.022	-0.12	0.17	-0.13
Leaf base angle (°)	0.47	0.73	**-0.25**	**-0.26**	0.052	0.079	0.11	0.041	0.039	-0.015	0.15	0.22	-0.052	-0.18	**-0.32**
Leaf tip angle (°)	0.25	0.49	0.035	-0.21	**-0.33**	-0.11	-0.21	-0.22	-0.14	-0.13	**0.25**	0.089	0.11	**-0.31**	0.046
Serrations (cm^-1^)	0.25	0.49	**-0.32**	-0.23	0.05	**0.28**	**0.25**	0.16	**0.25**	0.13	**0.27**	**0.3**	-0.23	-0.16	**-0.27**
Petiole length (mm)	0.27	0.53	**-0.31**	**-0.26**	-0.0077	**0.27**	**0.24**	0.19	0.18	0.15	**0.24**	**0.29**	-0.14	-0.18	**-0.31**
Petiole width (mm)	0.29	0.56	**0.24**	0.11	0.023	-0.18	**-0.24**	-0.24	-0.24	-0.22	**-0.21**	-0.22	0.17	0.11	**0.13**

H^2indiv^, broad-sense heritability of individual leaf measurements among three replicate blocks; H^2geno^, broad-sense heritability of the genotype mean across three blocks; FFD, frost free days; MAT, mean annual temperature; AST, annual summer temperature; MTCM, mean temperature of coldest month; MTWM, mean temperature of warmest month; MAP, mean annual precipitation; MSP, mean summer precipitation; CONT, continentality; ADI, annual dryness index; SDI, summer dryness index. Significant correlations of leaf traits with geo-climactic variables (Pearson) after correction for multiple testing are indicated in bold.

Most traits showed significant correlation with at least one geo-climatic variable (**Table 1**), suggesting that adaptation to environmental conditions has played a role in the evolution of leaf morphology in *P. balsamifera.* For example, serration density and petiole length were negatively correlated with latitude and positively with mean annual temperature (MAT), and leaf length was negatively correlated with summer dryness index (SDI) (**Table 1**). These observations contrast a study of Swedish quaking aspen, *Populus tremula*, which detected no correlation with leaf shape and geographical or climatic factors (Mähler et al., 2020).

### Ancestral population structure in Canadian *P. balsamifera*

We previously reported provenances originating from Roseville, Prince Edward Island (ROS) and Calgary, Alberta (CGY) as *P. balsamifera* × *P. deltoides* and *P. balsamifera × P. angustifolia* hybrids, respectively (Champigny et al., 2019). These verified hybrids are characterized by extremes of leaf morphology that distinguish them from *P. balsamifera*. Leaves of the ROS hybrid, for example, are significantly longer, wider, and with longer petioles (**Supplementary Figure S2**).

Genome-wide association studies conducted in our laboratories using *P. trichocarpa* populations tested kinship-based and PCA-based methods of controlling for population stratification among genotypes (Porth et al., 2013; McKown et al., 2014, 2018, 2019). GWAS of stomatal traits, for example, indicated that structure determination based on principal component analysis (Price et al., 2006) resulted in models with better fit (McKown et al., 2019). Population structure analyses of 313 P*. balsamifera* genotypes were performed to detect remaining hybridization events and gain further insight into the cryptic population structure of balsam poplar in the AgCanBaP collection.

**Figure 2:**
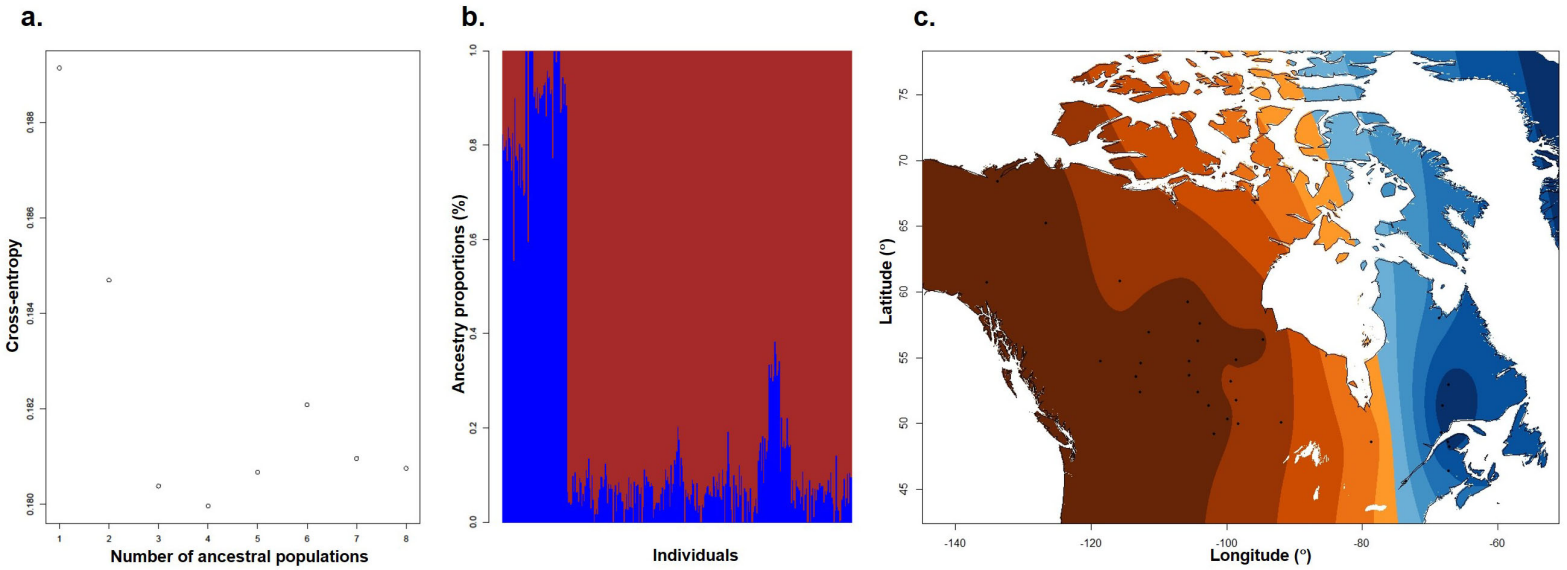
Genetic population structure among 313 P*. balsamifera* genotypes. Admixture analyses were conducted on a subset of 500,000 MAF and LD-filtered SNPs. **(a)** Cross-entropy using cluster solutions K = 1 to 8. **(b)** Barplot of ancestry coefficients for cluster solution K = 2. **(c)** Geographical projection of interpolated admixture estimates for the K = 2 solution.

*P. balsamifera* genotypes in the AgCanBaP collection were genome sequenced and SNPs identified and filtered (excluding ROS and CGY provenances) to select for a total of 5,641,729 bi-allelic SNPs present at a minor allele frequency greater than 5%. Admixture analysis detected two ancestral *P. balsamifera* populations within the 313 genotypes analyzed, an eastern deme and a western/northern deme (**Figures 2a-c**). Hierarchical clustering also identified a significant division between eastern and western/northern provenances (**Supplementary Figure S3**). We detected no evidence of hybridization among the remaining 313 genotypes and no evidence of clonal genotypes. Detection of eastern and western demes is consistent with a previous study of North American *P. balsamifera* population structure, which also identified an additional northern ancestral population present in the Yukon and Alaska (Keller et al., 2010). The present study includes only a single Alaskan provenance (Denali National Park - DEN), which likely explains the inability to resolve a distinct northern deme.

### Genetic architecture of leaf morphology in *P. balsamifera* revealed by FarmCPU

Following our earlier genome-wide association studies in *P. trichocarpa* (McKown et al., 2014, 2018, 2019), we attempted to identify SNP-leaf trait associations with a univariate mixed linear model using GEMMA (Zhou and Stephens, 2014). to estimate variance components. In this set of modelling experiments, structure correction based on kinship matrices, PCA, and a combination of the two as fixed effects were attempted. We found that SNP-trait models fit using the single locus mixed linear model are characterized by poor fit and no significant associations were detected (**Supplementary Figure S4**). A lack of significant associations using the same mixed linear model was reported in a leaf morphology study conducted in *Populus tremula* (Mähler et al., 2020).

Several algorithms for association mapping have been developed to overcome the limitations of single locus models whose statistical power decreases rapidly as the number of loci controlling variance increases. Recent studies examined the performance of multilocus GWAS methods with respect to traits with contrasting heritabilities and genetic architectures in species with differing rates of linkage disequilibrium. The Fixed and random model Circulating Probability Unification (FarmCPU) algorithm (Liu et al., 2016) obtained best results for modestly complex traits (Miao et al., 2019) and consistently identified associations in closest proximity to genes known to influence the studied traits (Kaler et al., 2020).

**Figure 3:**
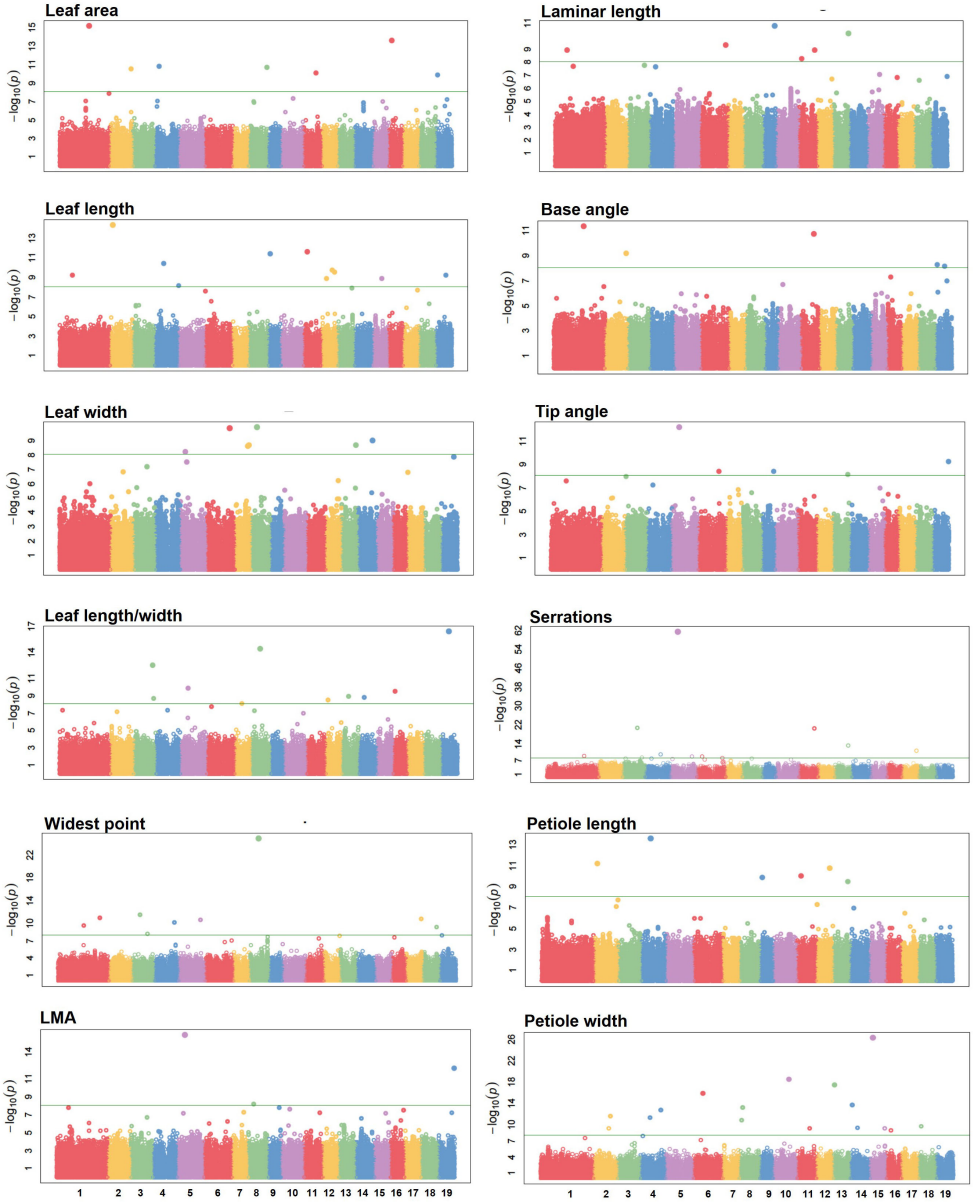
Manhattan plots of marker associations with *P. balsamifera* leaf morphological traits computed by FarmCPU. X axis – chromosomal position. Y axis – probability of association. Horizontal lines indicate the Bonferroni multiple testing threshold (*P* < 8.8 × 10^-9^) employed for determining significant SNP-trait associations.

Using FarmCPU, significant SNP-trait associations meeting the stringent Bonferroni threshold for multiple testing, *P* < 8.8 × 10^-9^, were identified for each of the 12 leaf morphological traits (**Figure 3**; **Table 2**). The number of loci detected ranged from 3 for LMA to 16 for petiole width. SNPs correlated with potential quantitative trait nucleotides (QTNs) are eliminated during fixed and random iterations of the FarmCPU algorithm (Liu et al., 2016). For this reason, marker-trait associations are detected as single SNPs in contrast to the traditional “peaks” including correlated SNPs typical of other GWAS methods (**Figure 3**).

**Table 2:** Loci detected as significant SNP-trait associations with *P. balsamifera* leaf morphological traits.

Trait	SNP	*P* value	Gene model	TAIR10	*Arabidopsis* protein	Function
Length/width						
	Chr19:7425655_C/T	3.97E-17	Potri.019G049700	AT3G03050		cellulose synthase-like D3
	**Chr08:8855438_C/A**	**3.83E-15**	**Potri.008G133400**	**AT3G17860**	**JAI3,JAZ3,TIFY6B**	**jasmonate-zim-domain protein 3**
	Chr03:18572848_T/A	3.10E-13	Potri.003G177900	AT4G13690		
	Chr05:7411732_G/T	1.34E-10	Potri.005G097800	AT1G31320		LOB domain-containing protein 4
	Chr16:1956212_G/C	3.28E-10	Potri.016G033700	AT5G05130		SWI/SNF-related
	Chr13:6802047_C/T	1.24E-09	Potri.013G078300	AT5G62030		- 2-(3-amino-3-carboxypropyl)histidine synthase
	Chr14:5764539_T/A	1.64E-09	Potri.014G071100	AT2G45440	DHDPS2	dihydrodipicolinate synthase
	Chr03:19559607_A/G	2.17E-09	Potri.003G191100	AT3G12400	ATELC,ELC	Ubiquitin-conjugating enzyme/RWD-like protein
	Chr12:2220528_T/A	3.26E-09	Potri.012G026200			
	Chr07:6526253_C/T	8.57E-09	Potri.007G060400	AT5G66055		ankyrin repeat protein
Leaf width						
	Chr08:5769920_C/T	1.18E-10	Potri.008G092000	AT1G13950		eukaryotic elongation factor 5A-1
	Chr06:22376711_T/G	1.32E-10	Potri.006G208400	AT2G36090		F-box family protein
	Chr14:14018605_T/C	9.91E-10	Potri.014G173100	AT3G25530	ATGHBDH,GHBDH,GLYR1	glyoxylate reductase 1
	Chr13:13931054_T/A	2.01E-09	Potri.013G126800	AT1G05710		basic helix-loop-helix (bHLH) DNA-binding
	Chr07:13490447_G/C	2.01E-09	Potri.007G112600	AT5G64330	JK218,NPH3,RPT3	Phototropic-responsive NPH3 family protein
	Chr07:12415998_T/C	2.53E-09	Potri.007G097900	AT5G63770	ATDGK2,DGK2	diacylglycerol kinase
	Chr05:4650704_A/G	5.86E-09	Potri.005G064500	AT5G09280		Pectin lyase-like superfamily protein
Leaf area						
	**Chr01:29484734_G/A**	**6.85E-16**	**Potri.001G289200**	**AT1G50030**	**TOR**	**target of rapamycin**
	Chr16:3308508_T/A	2.52E-14	Potri.016G051700	AT1G51200		A20/AN1-like zinc finger family protein
	Chr04:4479607_T/C	1.47E-11	Potri.004G056300	AT5G45890	SAG12	senescence-associated gene 12
	Chr08:18315884_A/T	2.05E-11	Potri.008G219300	AT3G14067		Subtilase family protein
	**Chr02:20806027_A/C**	**3.05E-11**	**Potri.002G221100**	**AT2G05160**		**CCCH-type zinc finger family**
	**Chr11:12377032_C/T**	**7.53E-11**	**Potri.011G101400**	**AT1G78510**	**SPS1**	**solanesyl diphosphate synthase 1**
	Chr19:1524533_T/G	1.33E-10				
Leaf length						
	**Chr02:2549504_C/T**	**4.63E-15**	**Potri.002G039700**	**AT1G04360**		**RING/U-box superfamily protein**
	**Chr11:3619189_T/G**	**2.47E-12**	**Potri.011G042500**	**AT4G05020**	**NDB2**	**NAD(P)H dehydrogenase B2**
	**Chr09:2180391_C/T**	**4.08E-12**	**Potri.009G012900**	**AT5G60550**	**ATSNAK1,GRIK2**	**geminivirus rep interacting kinase 2**
	**Chr04:9021110_T/G**	**3.68E-11**	**Potri.004G102700**	**AT1G52640**		**Pentatricopeptide repeat (PPR) superfamily protein**
	Chr12:10001319_T/A	1.85E-10	Potri.012G074800	AT1G62020		Coatomer, alpha subunit
	**Chr12:12502840_G/A**	**2.88E-10**	**Potri.012G099700**	**AT3G27740**	**CARA**	**carbamoyl phosphate synthetase A**
	Chr01:12706601_G/A	5.89E-10	Potri.001G154000			
	Chr19:9841873_C/T	6.13E-10	Potri.019G065600	AT3G10840		alpha/beta-Hydrolases superfamily protein
	Chr15:8507200_G/A	1.28E-09	Potri.015G062100	AT5G61410	EMB2728,RPE	D-ribulose-5-phosphate-3-epimerase
	Chr12:4426750_T/A	1.34E-09	Potri.012G047400	AT1G48600	PMEAMT	S-adenosyl-L-methionine-dependent methyltransferase
	Chr04:23900731_C/T	6.74E-09	Potri.004G232400	AT5G49760		Leucine-rich repeat protein kinase family protein
Petiole length						
	**Chr04:9021110_T/G**	**2.60E-14**	**Potri.004G102700**	**AT1G52640**		**Pentatricopeptide repeat (PPR) superfamily protein**
	**Chr02:2549504_C/T**	**6.08E-12**	**Potri.002G039700**	**AT1G04360**		**RING/U-box superfamily protein**
	**Chr12:12502840_G/A**	**1.67E-11**	**Potri.012G099700**	**AT3G27740**	**CARA**	**carbamoyl phosphate synthetase A**
	**Chr11:3619189_T/G**	**9.70E-11**	**Potri.011G042500**	**AT4G05020**	**NDB2**	**NAD(P)H dehydrogenase B2**
	**Chr09:2180391_C/T**	**1.32E-10**	**Potri.009G012900**	**AT5G60550**	**ATSNAK1,GRIK2**	**geminivirus rep interacting kinase 2**
	Chr13:14020710_C/A	3.16E-10	Potri.013G127600			
Petiole width						
	Chr15:2506679_C/T	5.87E-27	Potri.015G031300	AT1G14700	PAP3	purple acid phosphatase 3
	Chr10:14797793_C/T	2.88E-19	Potri.010G133300	AT3G61460	BRH1	brassinosteroid-responsive RING-H2
	Chr13:1167987_C/A	3.13E-18	Potri.013G017800	AT5G51060	ATRBOHC,RBOHC,RHD2	NADPH/respiratory burst oxidase protein D
	Chr06:9112436_T/C	1.48E-16	Potri.006G115800	AT5G03730	AtCTR1,CTR1,SIS1	serine/threonine kinase
	Chr14:1690390_G/T	2.21E-14	Potri.014G017500	AT1G49890		Family of unknown function (DUF566)
	Chr08:3259266_A/T	6.31E-14	Potri.008G055100	AT3G55370	OBP3	OBF-binding protein 3
	Chr04:19331273_C/A	1.64E-13	Potri.004G174800	AT2G16730	BGAL13	beta galactosidase - glycosyl hydrolase family 35
	Chr02:15834266_A/T	2.42E-12	Potri.002G197400	AT4G02110		transcription coactivator
	Chr04:8945671_T/C	4.47E-12	Potri.004G102000	AT4G05160		AMP-dependent synthetase and ligase family protein
	Chr08:2336551_G/A	1.45E-11	Potri.008G041000	AT3G54890	LHCA1	photosystem I light harvesting complex gene 1
	Chr18:2421181_C/T	2.03E-10	Potri.018G031100	AT5G11420		Protein of unknown function, DUF642
	Chr14:6764297_T/C	3.29E-10	Potri.014G085900	AT4G29910	ORC5	origin recognition complex protein 5
	Chr11:11828047_A/T	4.05E-10	Potri.011G097100	AT1G53190		RING/U-box superfamily protein
	Chr02:14328008_T/C	4.15E-10	Potri.002G183000	AT5G63140	PAP29	purple acid phosphatase 29
	Chr15:13741693_G/A	4.18E-10	Potri.015G123800	AT4G08850		Leucine-rich repeat receptor-like protein kinase family
	Chr16:4265540_T/C	9.87E-10	Potri.016G061000	AT1G58170		Disease resistance-responsive (dirigent-like protein)
Serrations						
	**Chr05:7172823_C/G**	**5.18E-62**	**Potri.005G095500**	**AT5G23960**	**ATTPS21,TPS21**	**sesquiterpene synthase, homologue of *PtTPS5***
	Chr03:14107199_A/C	1.55E-21	Potri.003G117700	AT1G62960	ACS10	ACC synthase 10
	Chr11:14421158_T/A	3.43E-21	Potri.011G118800	AT1G53025		Ubiquitin-conjugating enzyme family protein
	Chr13:12847444_A/T	4.41E-14	Potri.013G115200	AT1G22710	ATSUC2,SUC2,SUT1	sucrose-proton symporter 2
	Chr17:13709745_A/T	9.73E-12	Potri.017G125200	AT2G01570	RGA,RGA1	GRAS family transcription factor family protein
	Chr04:14788284_G/A	2.72E-10	Potri.004G131600	AT5G46280	MCM3	Minichromosome maintenance (MCM2/3/5) family
	Chr01:35956986_C/G	1.82E-09	Potri.001G352200	AT4G18210	ATPUP10,PUP10	purine permease 10
	Chr06:4751887_G/A	1.98E-09	Potri.006G064700	AT5G33290	XGD1	xylogalacturonan beta-1,3-xylosyltransferase
	Chr05:20852999_C/A	2.16E-09	Potri.005G191400	AT1G43670		Inositol monophosphatase family protein
Laminar length						
	Chr09:11226456_T/C	1.66E-11	Potri.009G140800	AT1G47670		Transmembrane amino acid transporter family
	Chr13:15036899_G/T	6.40E-11	Potri.013G145400	AT4G28190	ULT1	Developmental regulator, ULTRAPETALA
	Chr06:25243977_G/T	4.96E-10	Potri.006G244600	AT4G32680		
	Chr01:11957391_A/T	1.16E-09	Potri.001G146800	AT5G45470		Protein of unknown function (DUF594)
	Chr11:15541543_C/T	1.17E-09	Potri.011G129900	AT5G54530		Protein of unknown function, DUF538
	Chr11:2702470_G/A	5.48E-09	Potri.011G033000	AT1G61660		basic helix-loop-helix (bHLH) DNA-binding superfamily
LMA						
	**Chr05:7170178_G/A**	**1.28E-16**	**Potri.005G095500**	**AT5G23960**	**ATTPS21,TPS21**	**sesquiterpene synthase, homologue of *PtTPS5***
	**Chr19:15459585_T/G**	**6.63E-13**	**Potri.019G128100**	**AT2G30360**	**CIPK11,PKS5,SIP4,SNRK3.22**	**SOS3-interacting protein 4**
	Chr08:5530719_T/C	6.94E-09	Potri.008G088200	AT2G03060	AGL30	AGAMOUS-like 30
Widest point						
	**Chr08:8850239_G/A**	**1.43E-25**	**Potri.008G133400**	**AT3G17860**	**JAI3,JAZ3,TIFY6B**	**jasmonate-zim-domain protein 3**
	Chr03:7686128_T/A	2.96E-12	Potri.003G051900	AT3G16175		Thioesterase superfamily protein
	Chr01:41351792_C/T	1.07E-11	Potri.001G394200	AT4G20010	OSB2,PTAC9	plastid transcriptionally active 9
	Chr17:14683298_A/C	1.66E-11	Potri.017G138500	AT3G54230	SUA	suppressor of abi3-5
	Chr05:21072076_C/T	2.29E-11	Potri.005G193400	AT1G76760	ATY1,TRX-Y1,TY1	thioredoxin Y1
	Chr04:19660431_G/A	5.80E-11	Potri.004G179200	AT1G05680	UGT74E2	Uridine diphosphate glycosyltransferase 74E2
	Chr01:25474293_C/T	2.03E-10	Potri.001G243600	AT3G46640	LUX,PCL1	Homeodomain-like superfamily protein
	Chr18:13648134_A/G	4.33E-10	Potri.018G110200	AT1G63270	ATNAP10,NAP10	non-intrinsic ABC protein 10
	Chr03:14998970_A/T	5.77E-09	Potri.003G130300	AT1G07350		RNA-binding (RRM/RBD/RNP motifs) family protein
Tip angle						
	**Chr05:7170186_T/G**	**6.60E-13**	**Potri.005G095500**	**AT5G23960**	**ATTPS21,TPS21**	**sesquiterpene synthase, homologue of *PtTPS5***
	**Chr19:15459585_T/G**	**5.57E-10**	**Potri.019G128100**	**AT2G30360**	**CIPK11,PKS5,SNRK3.22**	**SOS3-interacting protein 4**
	Chr06:20401868_C/G	3.84E-09	Potri.006G189100	AT4G02050	STP7	sugar transporter protein 7
	Chr09:11226456_T/C	3.92E-09	Potri.009G140800	AT1G47670		Transmembrane amino acid transporter family protein
	Chr13:14461475_G/T	7.06E-09	Potri.013G133700	AT1G03670		ankyrin repeat family protein
Base angle						
	**Chr01:29484734_G/A**	**4.68E-12**	**Potri.001G289200**	**AT1G50030**	**TOR**	**target of rapamycin**
	**Chr11:12377032_C/T**	**1.90E-11**	**Potri.011G101400**	**AT1G78510**	**SPS1**	**solanesyl diphosphate synthase 1**
	**Chr02:20806027_A/C**	**6.36E-10**	**Potri.002G221100**	**AT2G05160**		**CCCH-type zinc fingerfamily protein with RNA-binding domain**
	Chr19:1524533_T/G	5.21E-09	Potri.019G013700	ATCG00020		photosystem II reaction center protein A
	Chr19:8790551_G/A	7.07E-09	Potri.019G056400	AT3G56290		

Chromosomal position in the *P. trichocarpa* v.3.0 reference genome, *P*-value, closest gene model, and best BLAST match to an *A. thaliana* TAIR10 gene model are indicated.

Our expectation of GWAS using correlated leaf morphological traits was that causative polymorphisms will explain phenotypic variance in multiple traits. For example, total leaf length is a linear combination of laminar length and petiole length traits (**Figure 1a**), implying that alleles explaining variance in leaf length influence either the length of the petiole or the length of the leaf blade. This expectation was borne out in the GWA results. Five of six polymorphisms detected as significant associations explaining variance in petiole length were also identified as associations explaining variance in total leaf length (**Table 2**). A total of 94 significant associations were detected, with 70 associations unique to a single trait and 24 were detected in association with more than one trait (**Table 2**). 73% of SNPs identified are located within an intergenic region, 5.3% within a predicted UTR, 6.4% within an intron, and 13% within the coding sequence of a poplar gene model (**Supplementary Table S1**).

The five shared SNPs identified by FarmCPU explaining variance in petiole length as well as leaf length are in closest proximity to gene models encoding a putative pentatricopeptide repeat superfamily protein, a RING/U-box superfamily member, carbamoyl phosphate synthetase A, NAD(P)H dehydrogenase B2, and an SnRK1 activating kinase (SnAK) (**Table 2**). These gene candidates have no obvious prior association with the regulation of petiole length, but few genetic mapping studies have examined this trait.

Of the 16 significant SNPs identified with respect to petiole width, 2 SNPs are located in closest proximity to gene models (*Potri.015G031300*, *Potri.002G183000*) encoding putative *P. balsamifera* purple acid phosphatases (**Table 2**). The PAP family is characterized by non-specific acid phosphatase activity, catalyzing the hydrolysis of inorganic phosphate (P_i_) from a variety of organic substrates. During phosphate starvation associated with nutrient-poor soils or leaf senescence, intracellular and secreted PAPs are upregulated to increase available stores of phosphorus (Tran et al., 2010). For example, *Arabidopsis* and rice orthologues of PAP26 play a crucial role in timing leaf senescence and remobilizing P_i_ from senescing to non-senescing leaves Robinson et al., 2012; Gao et al., 2017).

A polymorphism detected only in association with the leaf laminar length trait occurs in closest proximity to a gene model (*Potri.013G145400*) with highest similarity to ULTRAPETALA1, a developmental regulator of meristem patterning in *Arabidopsis* (**Table 2**). ULT1 is a trithorax group protein that plays pleiotropic roles in flower, reproductive, and leaf development by regulating the deposition of repressive histone modifications (Carles and Fletcher 2009). In *Arabidopsis*, ULT1 contributes to the patterning of the leaf adaxial/abaxial axis by promoting adaxial cell fates (Pires et al., 2015). Interestingly, we did not detect *Potri.013G145400* in association with the adaxial/abaxial trait LMA, but leaf laminar length, an aspect of proximal/distal polarity.

A group of associations implicates the TOR (Target Of Rapamycin) developmental pathway in the regulation of leaf morphology in balsam poplar. The most significant association explaining variance in leaf base angle and total leaf area occurs at position 29,484,734 on chromosome 1, located within an exon of the *P. balsamifera* TOR orthologue (*Potri.001G289200*). TOR is an ancient, evolutionarily conserved serine/threonine kinase, integrating nutrient and environmental signals to regulate organogenesis in all eukaryotes (McCready et al., 2020). In *A. thaliana*, TOR is expressed in primary meristematic regions (Ren et al., 2012) and its activation via glucose signaling is an important stimulator of cell proliferation (Xiong et al., 2013; Li et al., 2017). Knockdown of TOR expression has been shown to result in reduced cell size and smaller leaves (Deprost et al., 2007; Xiong and Sheen, 2012), while ectopic expression of TOR has been shown to be associated with larger epidermal cells and longer petioles (DeProst et al., 2007; Ahn et al., 2015).

TOR may regulate leaf development by interacting with pathways directly controlling cell division, for example by phosphorylation activation of E2Fa (Xiong et al., 2013). However, this highly pleiotropic protein also regulates nutrient transport (Ingargiola et al., 2020) including a role in affecting sugar transport through plasmodesmata (Brunkard et al., 2020), suggesting that TOR influences the size and shape of plant leaves by participating in multiple coordinated pathways.

In plants, TOR activity is regulated by glucose signaling via the activation of kinase complexes including the antagonistic Sucrose non-fermenting Related Kinases SnRK1 and SnRK2 (reviewed in K et al., 2019). In our study, we detected an association with tip angle and LMA (**Table 2**) linked to an orthologue of *A. thaliana CIPK11*, encoding a putative SnRK3 family member (*Potri.019G128100*). SnRK1 activity is itself regulated through phosphorylation by upstream SnRK1 Activating Kinases, SnAKs (Shen et al., 2009; Crozet et al., 2010; Glab et al., 2017). A SNP detected in association with total leaf length and petiole length traits is linked to a putative *P. balsamifera* SnAK1 orthologue, *Potri.009G012900* (**Table 2**). Cross talk among TOR, SnRK, and SnAK family members (including SnRK3) is well documented, participating in a common and ancient evolutionary mechanism of regulating TOR signaling (K et al., 2019).

### PbTPS5 is a candidate regulator of leaf morphology

The most significant marker-trait association with *P. balsamifera* leaf morphology is located at position 7,172,823 bp on chromosome 5. This SNP was detected at a *P*-value of 5.18 × 10–^62^ with respect to the density of leaf serrations (**Figure 3**; **Table 2**). The SNP identified is located within an intron of *Potri.005G095500*, a gene model whose closest amino acid sequence match in *A. thaliana* is TPS21, TERPENE SYNTHASE21 (**Table 2**). The most probable associations explaining variance in LMA and tip angle (**Figure 3**; **Table 2**) occur approximately 100 bp upstream of *Potri.005G095500*, likely located within its promoter regulatory region (**Figure 3**; **Table 2**).

In a phylogeny of terpene synthase genes expressed in *Populus trichocarpa, Potri.005G095500* is annotated as the sesquiterpene synthase *PtTPS5* (Irmisch et al., 2014). The leaf morphology-associated polymorphism is located in the balsam poplar orthologue of *PtTPS5* which we denote here as *PbTPS5*. *Arabidopsis TPS21* is expressed in floral organs and synthesizes β-caryophellene and λ-humulene as major components of the floral volatile mixture (Tholl et al., 2005). In poplar, gene models located on chromosome 19, namely *Potri.019G023100*, *Potri.019G000400*, and *Potri.019G016900* show higher sequence identity to the *Arabidopsis* caryophyllene/humulene synthase than does *PbTPS5*.

Terpenoids constitute a large and diverse class of primary and secondary metabolites playing roles in plant responses to herbivory and abiotic stress, as well as acting as volatile signal molecules (reviewed in Li et al., 2023; Singh and Sharma, 2015). However, their role in normal plant growth and development has received comparatively less attention. Terpenoids are synthesized from the 5-carbon monomers isopentenyl diphosphate (IPP) and dimethylallyl diphosphate (DMAPP). At least 38 members of the terpene synthase gene family are encoded in the *P. trichocarpa* genome, several having functions in response to caterpillar feeding (Irmisch et al., 2014). *Potri.005G095500* is a predicted sesquiterpene synthase, catalyzing the biosynthesis of 15-carbon terpenoids comprised of 3 isoprene units (Chen et al., 2011).

The sesquiterpene synthase-associated polymorphism detected by FarmCPU is present in the *P. balsamifera* population at a frequency of 10.1% (**Supplementary Table S2**). However, there exists a large degree of sub-population stratification in which the variant is dominant at a frequency of 77% within provenances of the eastern deme and a frequency of only 1.6% within provenances of the western deme. This significant correlation with cryptic population structure, as well as a lack of evidence for terpene synthases playing a role in leaf development raised the possibility that the *Potri.005G095500*-associated polymorphism is a statistical artifact.

The gene models located upstream of *Potri.005G095500* encode a putative mercaptopyruvate sulfurtransferase and a basic helix-loop-helix (bHLH) DNA-binding superfamily protein, likely a transcription factor with no obvious orthologue in *Arabidopsis*. These loci, encoded on opposite strands, are separated from *Potri.005G095500* by approximately 19 kb. Gene models located downstream of *Potri.005G095500* encode a putative aspartyl protease family protein, at a distance of 8 kb, and a protein with high similarity to *Arabidopsis SCI1* (*Stigma/Style Cell-cycle Inhibitor1*). Available data from expression and mutant studies in *Arabidopsis* and tobacco suggest SCI1 function is restricted to floral organ development (DePaoli et al., 2014).

We examined linkage disequilibrium (LD) relationships in the *P. balsamifera* genome as well as fine-scaled LD surrounding the detected SNP. In agreement with previous studies on *P. tremula* (Ingvarsson, 2008), linkage disequilibrium decays significantly at a distance of several hundred bases among genotypes in the AgCanBaP population (**Supplementary Figure S6a**). There is evidence of local LD in discrete blocks at a scale of several hundred bases within *Potri.005G095500*, but not at larger distances (**Supplementary Figure S6b**). Taken together, these analyses indicate that the causal polymorphism responsible for the association is most likely an allele affecting the activity of *Potri.005G095500*.

### Population structure-independent genomic prediction of *P. balsamifera* leaf morphology by GWADL

Genomic prediction is a statistical learning approach to breeding, involving the use of genetic markers to estimate the phenotype (so-called genotypic breeding value, GBV) of both observed and unobserved individuals (Meuwissen et al., 2001). By focusing resources on individuals predicted by genotyping to have the most desirable traits, the aim of genomic prediction is to increase genetic gains per unit time and cost. Genomic prediction is an enticing technology in breeding long-lived trees because of the timescale needed to phenotype biomass and wood quality traits over generations (Cappa et al., 2019).

The most commonly used genomic prediction models are the Best Linear Unbiased Predictions, BLUPs. Accurate representation of the population structure either by explicit pedigree (Henderson, 1985) or the genomic relationship matrix (GBLUP, Habier et al., 2007) is used to describe additive genetic relationships between individuals at QTL (Habier et al., 2013). Thus, the extremely popular GBLUP and computationally equivalent ridge regression BLUP (rrBLUP, Daetwyler et al., 2010) make predictions based on the ability to determine the relatedness of individuals in the test population in comparison to individuals in the training population. Several studies have demonstrated this dependence on genetic relatedness (Heslot et al., 2015; Voss-Fels et al., 2019; Zhang et al., 2017) and predictions sharply lose accuracy as unrelated individuals are added to a breeding population (Crossa et al., 2014; Edwards et al., 2019).

**Figure 4:**
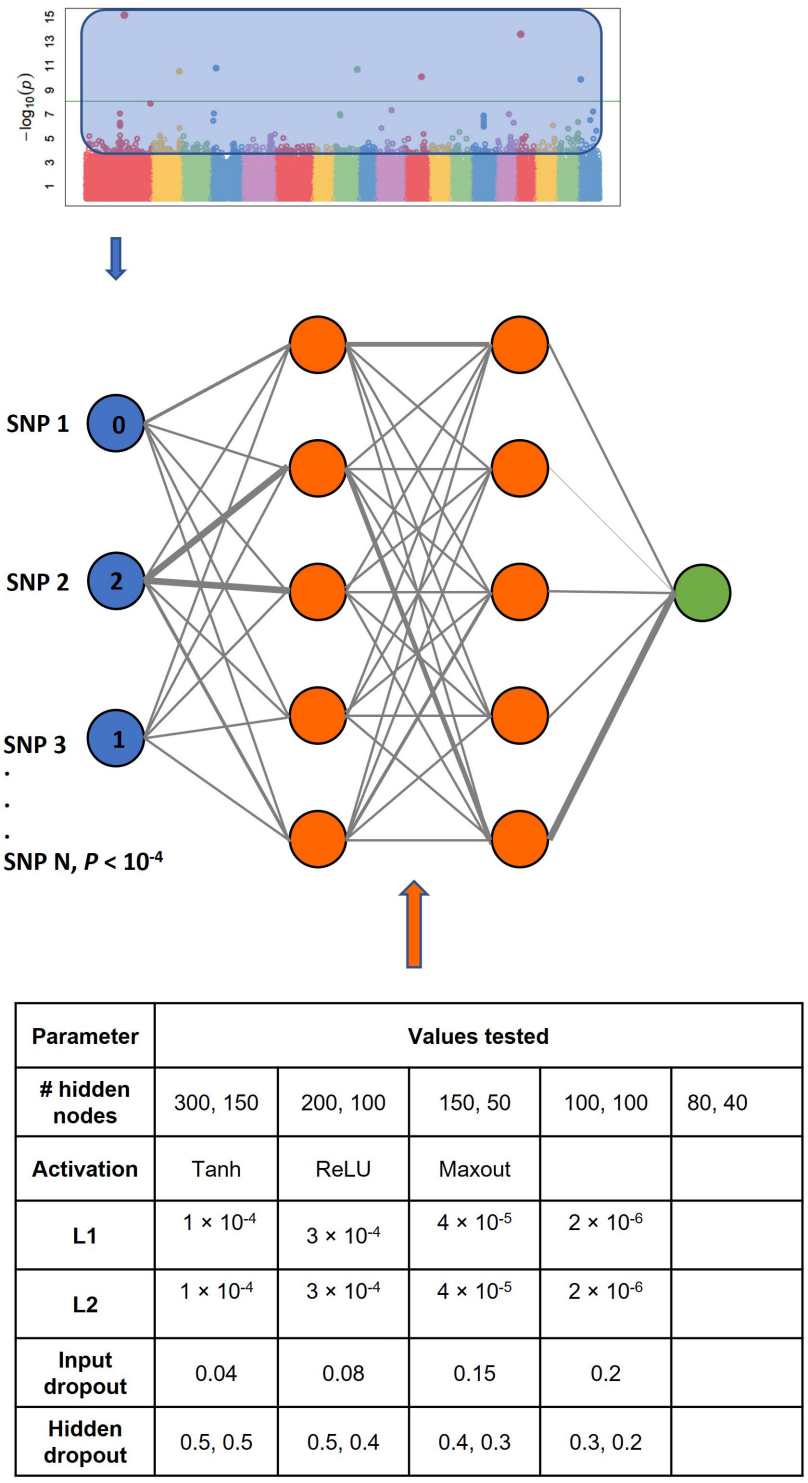
Schematic illustration of the GWADL (Genome-Wide Association enriched Deep Learning) method. Top – input layer consists of SNPs selected according to a prior probability of association by GWA. In this study, *P* < 10^-4^. Middle – neural network is a fully-connected fast forward neural network with two hidden layers. Output layer is a quantitative estimate of leaf morphological trait, or genomic breeding value, GBV. Bottom – Training with hyperparameter tuning optimizes activation function, ridge/lasso regularization, number of neurons in two hidden layers, dropout frequency, and network weights minimizing loss at output layer. Relative variable importance is calculated according to the algorithm of Gideon.

We investigated the use of GWAS *P*-value enrichment on genomic prediction of leaf morphological traits by constructing models using the explicit kinship-based GBLUP method, as well as fast-forward neural network models with two hidden layers (**Figure 4**). Our specific goal was to determine whether markers with a prior probability of association with leaf morphology can be used to produce models that are independent of genetic relatedness. The rationale behind this approach is that modern GWAS detects SNP markers whose probability of association is determined with correction for the underlying population structure. We sought to train a genomic prediction model to use *P*-value enriched SNPs in a manner independent of population structure.

In this study, GWAS identified a small number of loci known to be involved in leaf development (for example TOR and ULT1), but the majority of associations detected have no obvious relationship to leaf morphology. Hence, the false positive and false negative detection rate are unknown, and we had no prior expectation of how *P*-value enriched SNPs will perform in genomic prediction of leaf morphology.

**Figure 5:**
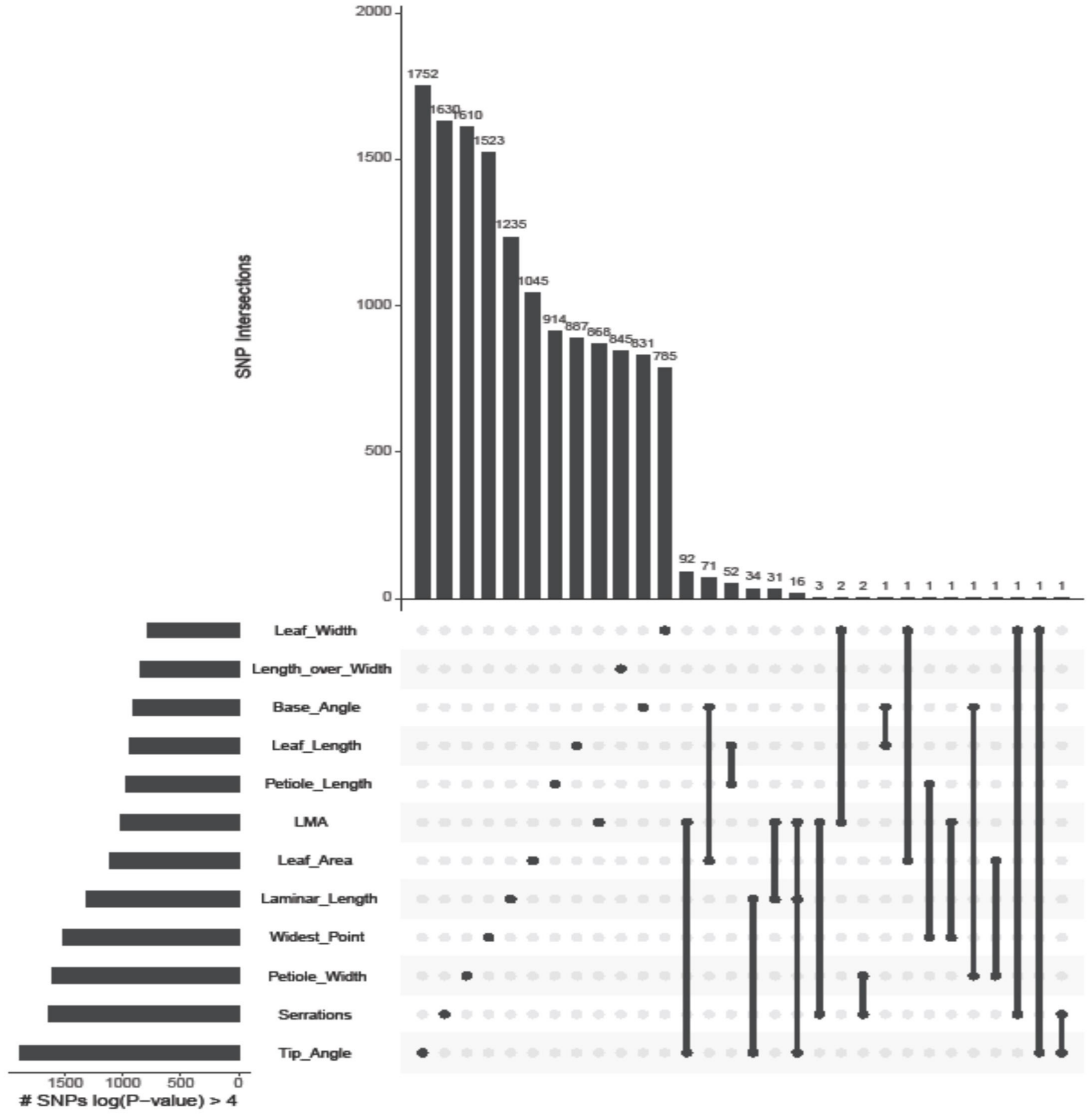
Intersections among SNPs selected at SNP-trait association probability *P* < 10^-4^. Intersections among subsets of SNPs are visualized as an UpSet plot sorted by size of intersection. Dark circles indicate sets included in the intersection; singleton dark circles indicate SNPs detected in association with a single trait and dark circles connected by lines indicate SNPs detected in association with more than one trait. Vertical bars denote membership in a set.

FarmCPU results indicated a degree of overlap in significant SNP-trait associations with respect to the correlated leaf traits. At *P* < 8.8 × 10^-9^, 26% of SNPs were detected as associations with more than one trait (**Figure 3**, **Table 2**). Visual inspection of Manhattan plots indicated that by using a *P*-value cutoff of 10^-4^, all SNPs with a probability of association above background level are included (**Figure 3**). At *P* < 10^-4^, the average number of SNPs selected per trait is 1,214, ranging from 790 SNPs associated with leaf width to 1,896 SNPs associated with tip angle. We conducted a set intersection analysis to gain insight into the distribution of SNPs selected below the Bonferroni threshold (**Figure 5**). Few loci are shared among leaf traits at lower levels of significance. Nearly all SNPs selected at *P* < 10–^4^ are unique to a single trait, and there are many intersections containing only the single SNP or pair of SNPs detected at *P* < 8.8 × 10^-9^ (**Figure 5**).

With respect to genomic prediction using SNPs at a threshold cutoff of *P* < 10^-4^, each trait model therefore used a nearly distinct set of markers, the exception being a very small number of SNPs detected as shared significant associations. As a comparison set to the SNPs selected at a GWAS *P-*value cutoff of 10^-4^, we selected equal numbers of widely distributed markers across the genome by choosing every *n^th^* polymorphism in the set of 5,641,729 bi-allelic SNPs identified by sequencing.

The present leaf morphology GWAS study is modest in scope, including only 313 different balsam poplar genotypes. In preliminary experiments, we investigated the use of an independent test set by randomly selecting and removing 70 genotypes. This expectedly had a deleterious effect on GWAS, losing approximately half of the significant associations. For this reason, our study did not include a test set and models were instead evaluated on the basis of an internal 5-fold cross validation.

GBLUP conducted using the sets of *P-*value enriched SNPs showed heterogenous results for predictions of the leaf morphology traits. The least performing prediction was for serration density, in which 25% ± 19% of heritable phenotypic variance was explained. The highest performing prediction was for LMA, in which 120% ± 7.3% of heritable variance was explained. Several of the trait predictions, including for LMA, exceeded the theoretical maximum given the heritabilities of the traits. Inflation of prediction accuracy based on cross-validation results alone is an expected feature of GBLUP models that lack regularization (i.e., a set of methods for reducing overfitting in ML models), and lower prediction accuracy is generally observed on test sets (as in Bermingham et al., 2015).

**Figure 6:**
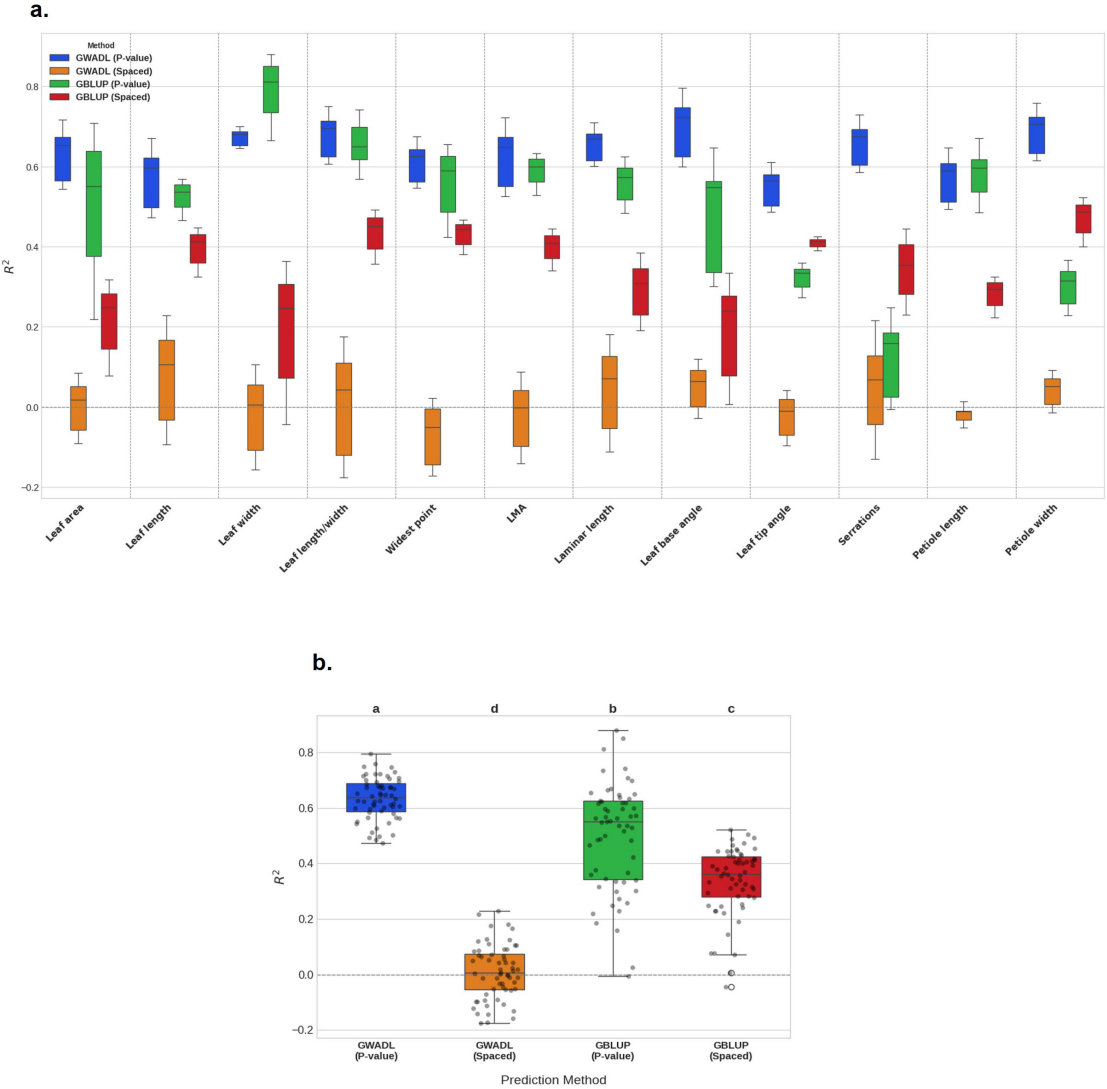
Genomic prediction of *P. balsamifera* leaf morphology. Prediction of 12 leaf morphological traits was conducted on 313 genotypes with 5-fold cross validation. **(a)** Genomic prediction accuracy presented as boxplots of trait prediction results (R^2^) on each cross-validation fold. Methods tested were: 1) GWADL using SNP inputs selected by GWA *P*-value enrichment, *P* < 10^-4^, 2) GWADL using an identical number of genomically-spaced SNPs selected by choosing n^th^ SNP in the genome, 3) and 4) GBLUP using the same *P*-value enriched or genomically-spaced SNPs as for GWADL. **(b)** Genomic prediction accuracy presented as boxplots of prediction results (R^2^) arranged by method used. Dots denote prediction accuracy for a trait cross-validation fold. Letters denote significant differences among genomic prediction methods according to Tukey’s HSD test.

We compared the GBLUP prediction accuracies for *P-*value enriched SNPs with models trained using equal numbers of well-spaced SNPs. In these experiments, prediction accuracy using genomically-spaced markers ranged from 26% ± 19% of heritable variance explained for leaf base angle to 84% ± 2.1% for LMA. *P*-value enrichment resulted in increased prediction accuracy for leaf area, leaf width, leaf length, length/width, and petiole length but decreased accuracy for leaf tip angle, serrations, and petiole width (**Figure 6a**). These results agree with previous studies showing that SNP enrichment for genomic prediction depends on factors such as the complexity of genetic architecture, heritability and relationship of selected SNPs to the linkage disequilibrium structure (reviewed in Xu et al., 2020).

Use of deep learning for genomic prediction has attracted interest in recent years because of the demonstrated ability of neural network models to capture non-additive, non-linear relationships of extreme complexity (reviewed in Montesinos-Lopez et al., 2021). Tantalizing clues in the literature suggest that the mechanism of genomic prediction used by neural networks is fundamentally different from BLUP and may be less affected by population structure. For example, it was observed that marker selection increased accuracy of neural network models, with poor results obtained using sets of randomly chosen SNPs (Bellot et al., 2018; Pérez-Enciso and Zingaretti, 2019). In our previous study modelling poplar phenotypes from natural patterns of DNA methylation, we also observed that deep learning models required feature selection and were not confounded by the large degree of population stratification in the experimental design (Champigny et al., 2019).

We reasoned that if genetic relatedness is represented by neural network models, then they should explain a significant proportion of heritable variance in leaf traits from the sets of SNPs that are well-spaced throughout the genome, as was the case for GBLUP. A high-performance computation approach was used to train 3,840 different fast-forward architectures for each trait, in which optimum values for the number of neurons in the two hidden layers, activation function, regularization, and dropout frequency were empirically determined (**Figure 4**). Using the genomically-spaced SNPs as input layers, the best performing models selected by cross-validation accuracy explained 1.2% ± 1.9% of phenotypic variance across leaf traits, clearly indicating no predictive capacity (**Figure 6b**). Thus, we trained 46,000 different deep learning architectures using 12 distinct sets of widely-spaced markers and traits and uncovered no evidence that the fast-forward neural networks represent or model LD structure.

The predictive capacity of genomically-spaced markers in deep learning genomic prediction was compared to models trained using *P*-value enriched SNPs as input, a strategy we call GWADL (Genome-Wide Association enriched Deep Learning, **Figure 4**). Across the 12 leaf morphology traits, the best performing GWADL models optimized by parameter tuning explained 108% ± 14% of heritable phenotypic variance, indeed for 8 of the 12 traits the heritability estimate is indistinguishable from the accuracy estimate of the model (**Figure 6a**; **Supplementary Table S2**). Although the neural networks trained in this study were optimized for lasso, ridge, and dropout regularization parameters (reviewed in Montesinos-Lopez et al., 2021) to combat overfitting, cross-validation results suggest a degree of overfitting. Nonetheless we generally observed less inflation compared to GBLUP (**Figure 6**).

Although tuning deep learning models requires greater computational resources than other machine learning methods, parameter optimization is crucial to the efficacy of GWADL. We compared the prediction accuracies of the best performing models with the worst performing models (simulating a “guess” of the parameters) in the model tuning strategies. All GWADL models trained in this study explained at least 55% of heritable variance in the leaf morphological traits. However, we found that parameter-optimized models explained, on average, 44% more heritable variance across the 12 leaf traits (**Supplementary Table S2**).

**Figure 7:**
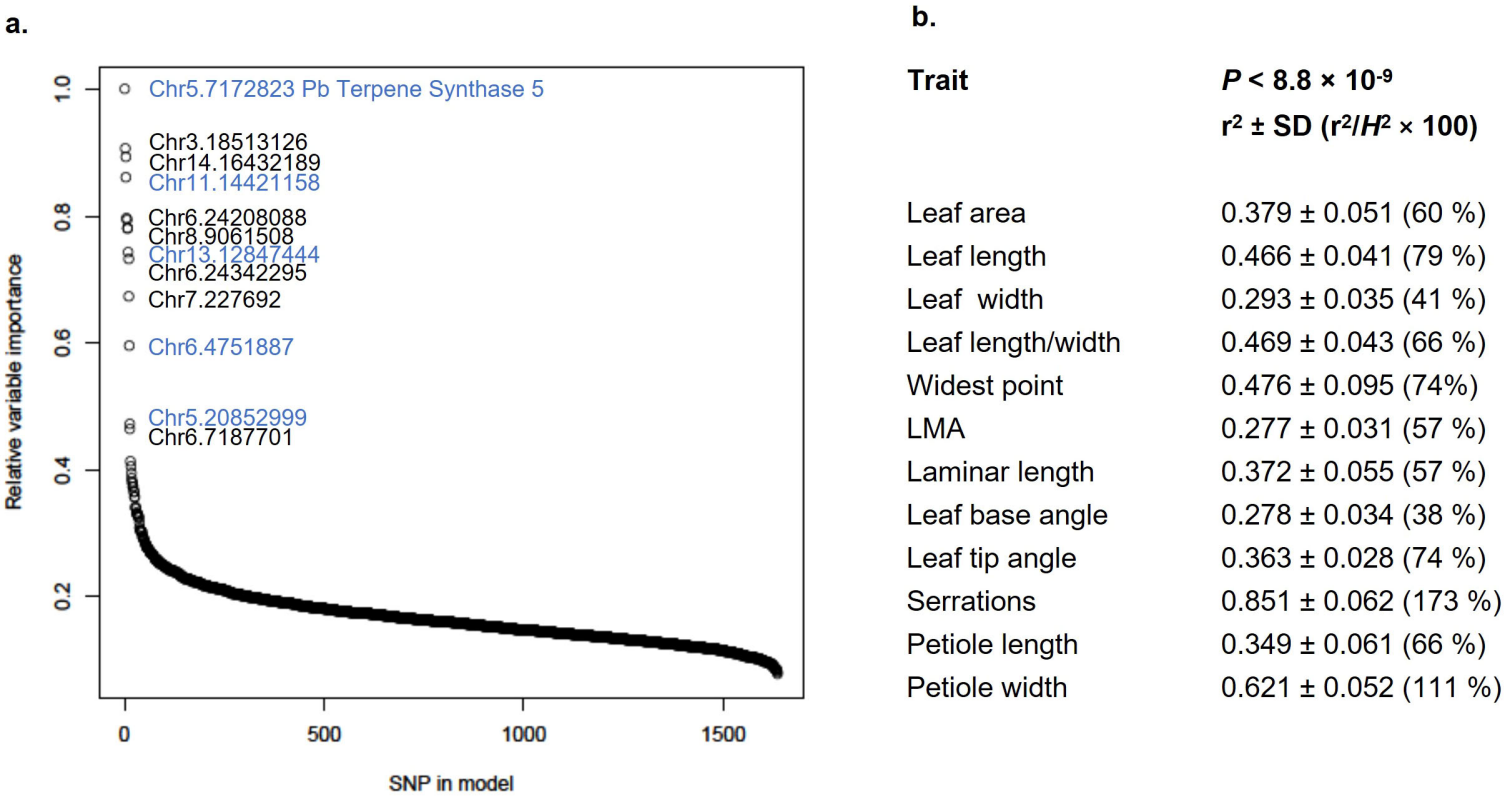
Variance in leaf morphology explained by GWADL. **(a)** Relative variable importance of SNPs used as input in the GWADL model predicting serration density (see **Figure 6b**). Blue colour indicates SNPs identified as significant associations by FarmCPU. **(b)** Phenotypic variance explained by GWADL models using only SNPs with *P*-value < 8.8 × 10^-9^.

These experiments demonstrate that, in contrast to the explicit kinship-based genomic prediction method GBLUP, fast-forward neural networks show a notable preference for SNP markers with a prior probability of association and selected against the underlying population structure. To gain further insight into the mechanism of GWADL, we conducted sensitivity analyses of the best-performing model for each trait. In sensitivity analysis, neural network weights are permuted in prescribed patterns and the effect on prediction accuracy is observed. The goal of this technique is to rank or score the relative importance of each variable in a neural network model (Pérez-Enciso and Zingaretti, 2019). Using a variable importance algorithm designed for fast-forward neural networks with two hidden layers (Gedeon, 1997), we found a highly skewed pattern of SNP importance. In each model, there were 10–20 SNPs whose variable importance is significantly higher than the background. Interestingly, the most important SNPs were highly enriched in markers detected as significant by FarmCPU. For example, in the GWADL model using 1,637 SNPs as input and predicting serration density, 5/9 markers detected by FarmCPU have highly elevated variable importance. Of particular interest, the *TPS5*-associated SNP is the most influential variable in the model and with the highest obtainable variable importance score (**Figure 7a**).

Results showing that genomically-spaced SNPs could not be used for deep learning genomic prediction (**Figure 6b**), as well as the variable importance of SNPs identified by FarmCPU (**Figure 7a**), suggest that uncharacterized genes detected by FarmCPU in association with leaf morphology may be genuine, rather than artifacts of population structure. We estimated the proportion of phenotypic variance in leaf morphological traits by GWADL using only the 3–16 SNPs detected as significant associations. Heritable variance explained ranged from 28% for leaf base angle to 85% for serration density (**Figure 7b**), results comparable to GBLUP using approximately 1,000 markers. Taken together, our results demonstrate that multilocus GWAS and deep learning can be used together for highly accurate, *de novo* genomic prediction of complex traits. Analyses of the GWADL method point toward a mechanism independent of genetic relatedness, thus it may help address practical breeding problems involving population structure and LD decay over generations.

### Exogenously applied sesquiterpenes significantly affect poplar leaf development

Using non-linear, non-additive, non-parametric neural networks to make marker-trait associations, we found that *Potri.005G095500*-associated SNPs are the most important predictors of tip angle, LMA, and serration density in agreement with FarmCPU results. Furthermore, our analyses provide evidence that this SNP was detected due to marker effect and independently of population structure, suggesting that *PbTPS5* has a major effect on leaf morphology and leading us to examine this hypothesis in greater detail.

Heterologous expression experiments demonstrated that poplar TPS5 is a sesquiterpene alcohol synthase, its minor products identified as elemol and β-eudesmol (Irmisch et al., 2014), and its major products identified as the novel sesquiterpenes (1S,5S,7R,10R)-Guaia-4(15)-en-11-ol and (1S,7R,10R)-Guaia-4-en-11-ol (Lackus et al., 2021). Transcript abundance of *PtTPS5* is upregulated 5-fold in response to caterpillar feeding on leaves (Irmisch et al., 2014) and approximately 150-fold in poplar roots in response to *Pseudomonas cactorum* infection (Lackus et al., 2021).

A small number of correlational studies have provided clues that sesquiterpenes can act as inhibitors of growth and development *in planta.* In sunflower, for example, exogenous application of sesquiterpenes was shown to antagonize auxin-dependent growth (Spring et al., 2020). In contrast, a large body of pharmacological research aimed at identifying plant-derived anti-cancer compounds has revealed that dozens of sesquiterpenes induce apoptosis in human cancer cells (reviewed in Akrivou et al., 2018; Bosco and Golsteyn, 2017; Hsu et al., 2024). The minor products of TPS5, elemol and eudesmol, as well as β-caryophyllene, the main product of *Arabidopsis* TPS21, have each been shown to arrest the cell cycle leading to cell death (Rezaieseresht et al., 2020; Kotawong et al., 2020; Park et al., 2011; Yu et al., 2021). It has been suggested that terpene synthases are essential for normal growth and development, potentially by modulating the cell cycle which remains poorly attested in plants (Akrivou et al., 2018; Bosco and Golsteyn, 2017).

**Figure 8:**
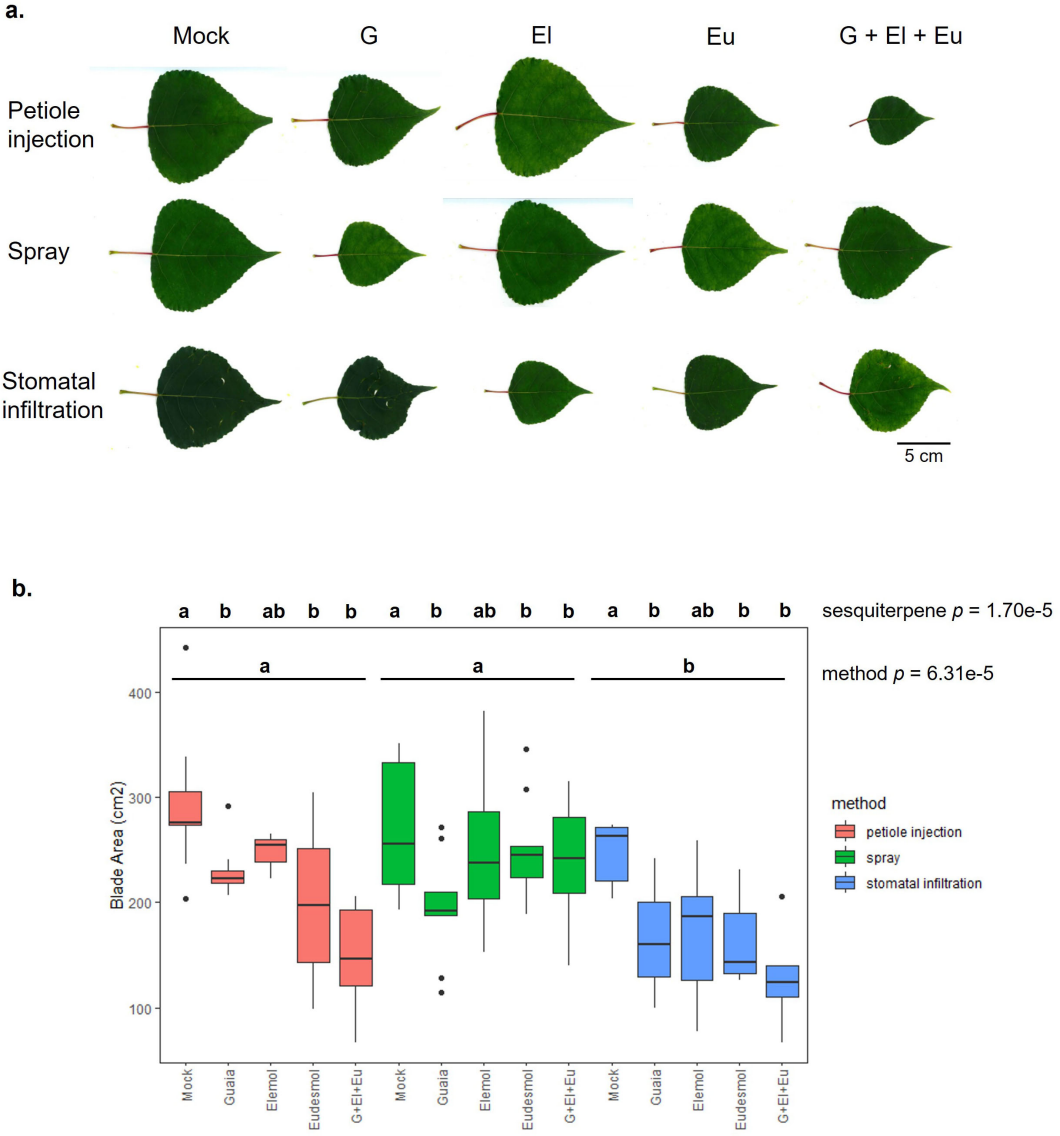
Effects of exogenously applied sesquiterpenes on poplar leaf development. **(a)** Representative images of sesquiterpene-treated Okanese leaves at maturity. **(b)** Boxplots of leaf blade area as a function of sesquiterpene treatment and application method. Letters denote significant differences according to Tukey’s HSD test. G: 1αH,5αH-G PbTPS5uaia-6-ene-4β,10β-diol, El: α, β -elemolic acid, Eu: β-eudesmol.

To investigate the role of *PbTPS5* in leaf development, we used exogenous application methods to examine the effects of sesquiterpene products of *PbTPS5* on developing hybrid poplar leaves in greenhouse experiments. The poplar genotype used (var. “Okanese”) is a *P.×’Walker’ and P.× petrowskyana* cross (Schroeder et al., 2013). The major products of poplar TPS5, namely (1S,5S,7R,10R)-Guaia-4(15)-en-11-ol and (1S,7R,10R)-Guaia-4-en-11-ol, could not be sourced commercially or from other laboratories. Therefore, we used a related sesquiterpene alcohol, 1αH,5αH-Guaia-6-ene-4β,10β-diol, identified in *Alisma orientale* (Ma et al., 2016), in addition to the TPS5 minor products elemol and eudesmol. Moreover, we used three application methods (petiole injection, leaf spray and stomatal infiltration) to test the effect of 50 *u*M of each sesquiterpene individually, as well as a combination of elemol, eudesmol, and 1αH,5αH-Guaia-6-ene-4β,10β-diol on developing Okanese leaves (**Figure 8**, **Supplementary Table S3**). Upon maturity, leaf size and shape were statistically analyzed by 2-way ANOVA using sesquiterpene treatment and application method as factors.

Observations of sesquiterpene-treated leaves demonstrated that, at 50 *u*M concentration, none of the treatments resulted in obvious necrosis or immediate cytotoxicity. We occasionally observed mechanical damage resulting from the stomatal infiltration method, but otherwise developing leaves remained healthy until maturity (**Figure 8a**). ANOVA analyses of the treated leaves demonstrated highly significant effects of both sesquiterpene (*P* = 1.70e^-5^) and application method (*P* = 6.31e^-5^) on blade area (**Figure 8b**). Exogenous application of eudesmol, 1αH,5αH-Guaia-6-ene-4β,10β-diol, as well as the combination treatment, resulted in significantly smaller mature leaves. The effect of elemol on leaf size was marginal according to Tukey’s HSD test. Leaves treated with sesquiterpenes using the stomatal infiltration method were significantly smaller than leaves treated by spraying or petiole injection (**Figure 8b**). Although sesquiterpene-treated leaves were smaller than controls, treatments did not appear to affect a specific axis of leaf development. For example, treated leaves exhibiting smaller blade area also had significantly smaller blade perimeter, length, and width (**Supplementary Table S3**). Petiole injection of the combined sesquiterpene solution resulted in narrower petioles (**Supplementary Table S3**). Our leaf development experiments show notable congruities with mammalian cell culture studies in that the sesquiterpene treatments exhibited no obvious non-specific cytotoxic effects, but nonetheless resulted in reduced rates of cell proliferation. Taken together, findings from classical GWAS, machine learning, as well as biological sesquiterpene treatments strongly implicate PbTPS5 in a novel role regulating leaf morphology.

In the context of our leaf development study, *in vitro* pharmacological studies are of additional interest because for many sesquiterpenes, including β-caryophyllene, cell cycle arrest and apoptosis is induced by downregulating mammalian TOR signaling (Park et al., 2011; Yu et al., 2021). The highly significant, reproducible detection of TPS5, TOR, and other putative members of the TOR pathway in our leaf morphology GWAS raises the possibility that PbTPS5 is an important regulator of leaf development through its participation in the TOR developmental pathway in poplar.

## Conclusions

By examining genetic variation, we elucidated how poplar leaf shape and size may have evolved along with climate adaptation. The ancient, evolutionarily conserved TOR signaling pathway, which integrates environmental and nutrient signals to direct growth and development, appears to be central to leaf morphology variation in *P. balsamifera*. Furthermore, we present new evidence of sesquiterpene synthase (TPS5) function in regulating leaf size and shape, providing a long-suspected example of terpene synthase activity contributing to plant development. Lastly, detection of natural alleles, as well as efficient genomic prediction models, lay the foundation for increasing *P. balsamifera* performance by optimizing leaf morphology.

## Data Availability

The datasets presented in this study can be found in online repositories. The names of the repository/repositories and accession number(s) can be found in the article/**Supplementary Material**.

## Supplementary material

Supplementary Figure 1 Intra-tree variation in leaf morphology in *P. balsamifera*. Representative photographs illustrating variation in leaf morphology among leaves sampled from a single tree.

Supplementary Figure 2 Significant differences in leaf morphology among *P. balsamifera* provenances. Boxplots of *P. balsamifera* leaf morphological traits after 4 years of growth in the Indian Head common garden. Observations are grouped by provenance and letters indicate significant differences among provenances according to Tukey’s HSD test.

Supplementary Figure 3 Hierarchical clustering analysis of population structure in 313 Canadian *P. balsamifera* genotypes. Hierarchical clustering conducted on 500,000 randomly selected SNPs using the Ward clustering method. Rectangles surround a single division supported at 0.95 level of significance. P-values denoted in green are estimated as bootstrap probabilities (bp) calculated using 1000 bootstrap iterations. P-values denoted in red are approximately unbiased (au).

Supplementary Figure 4 Representative GWAS results using GEMMA linear mixed model. GWA was conducted on the leaf length/width trait using the GEMMA univariate mixed linear model. Kinship matrix was obtained using the method of VanRaden and a PCA covariate (PC1) associated with longitude (East-to-West population stratification) was included to control for cryptic population structure. Top – QQ plot. Bottom – Manhattan plot.

Supplementary Figure 5 QQ-plots illustrating fit of GWAS models conducted by FarmCPU.

Supplementary Figure 6 Linkage disequilibrium in *P. balsamifera*. **(a)** Linkage disequilibrium decay plot illustrating pairwise correlation between SNPs as a function of genomic distance. **(b)** Linkage disequilibrium plot illustrating correlations among SNPs detected in *PbTPS5*. Correlations were calculated as *r*^2^. Blue colour indicates the SNP identified by GWAS.
