# Supplementary figures and images for: TPS5 and TOR signaling components are determinants of Populus balsamifera leaf morphology
Source: Front Plant Sci. 2025 Dec 4;16:1683866. doi: 10.3389/fpls.2025.1683866 (PMC12713574; doi:10.3389/fpls.2025.1683866)

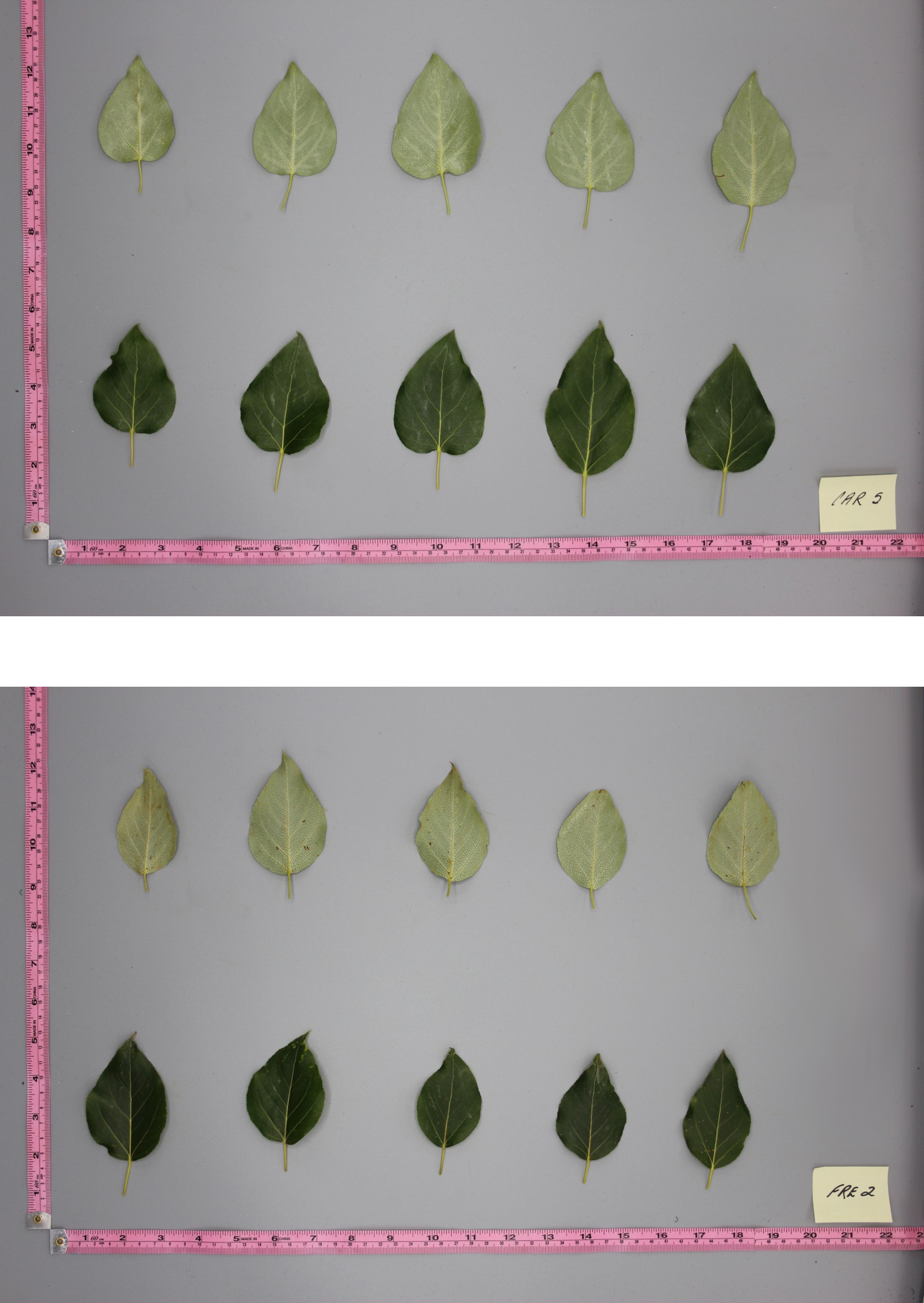

Supplement: Supplementary Figure 1 — Intra-tree variation in leaf morphology in P. balsamifera. Representative photographs illustrating variation in leaf morphology among leaves sampled from a single tree. [file Image1.jpg]

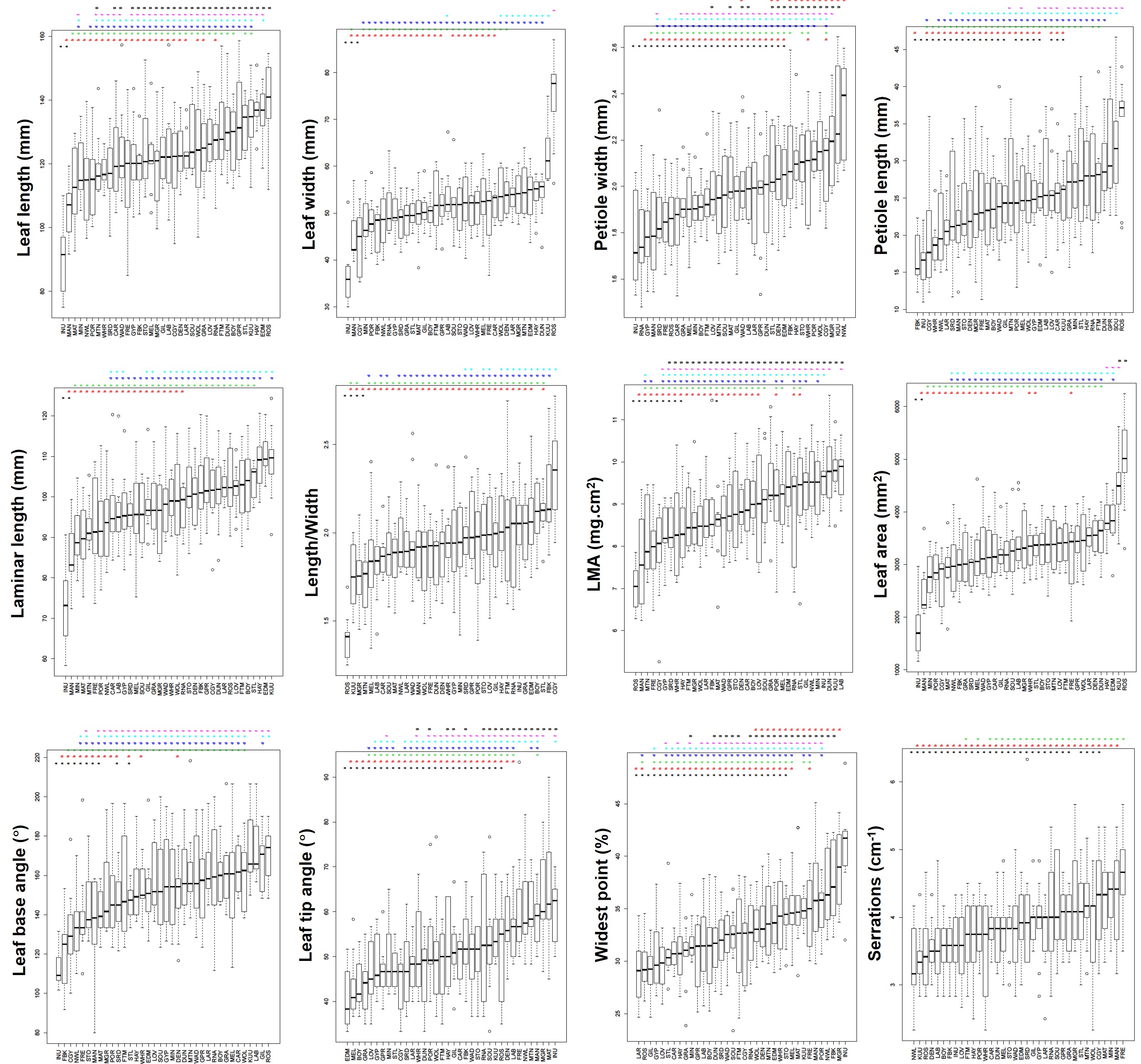

Supplement: Supplementary Figure 2 — Significant differences in leaf morphology among P. balsamifera provenances. Boxplots of P. balsamifera leaf morphological traits after 4 years of growth in the Indian Head common garden. Observations are grouped by provenance and letters indicate significant differences among provenances according to Tukey’s HSD test. [file Image2.jpg]

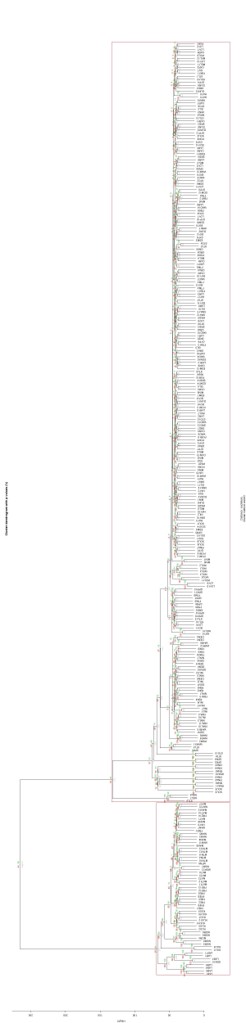

Supplement: Supplementary Figure 3 — Hierarchical clustering analysis of population structure in 313 Canadian P. balsamifera genotypes. Hierarchical clustering conducted on 500,000 randomly selected SNPs using the Ward clustering method. Rectangles surround a single division supported at 0.95 level of significance. P-values denoted in green are estimated as bootstrap probabilities (bp) calculated using 1000 bootstrap iterations. P-values denoted in red are approximately unbiased (au). [file Image3.jpg]

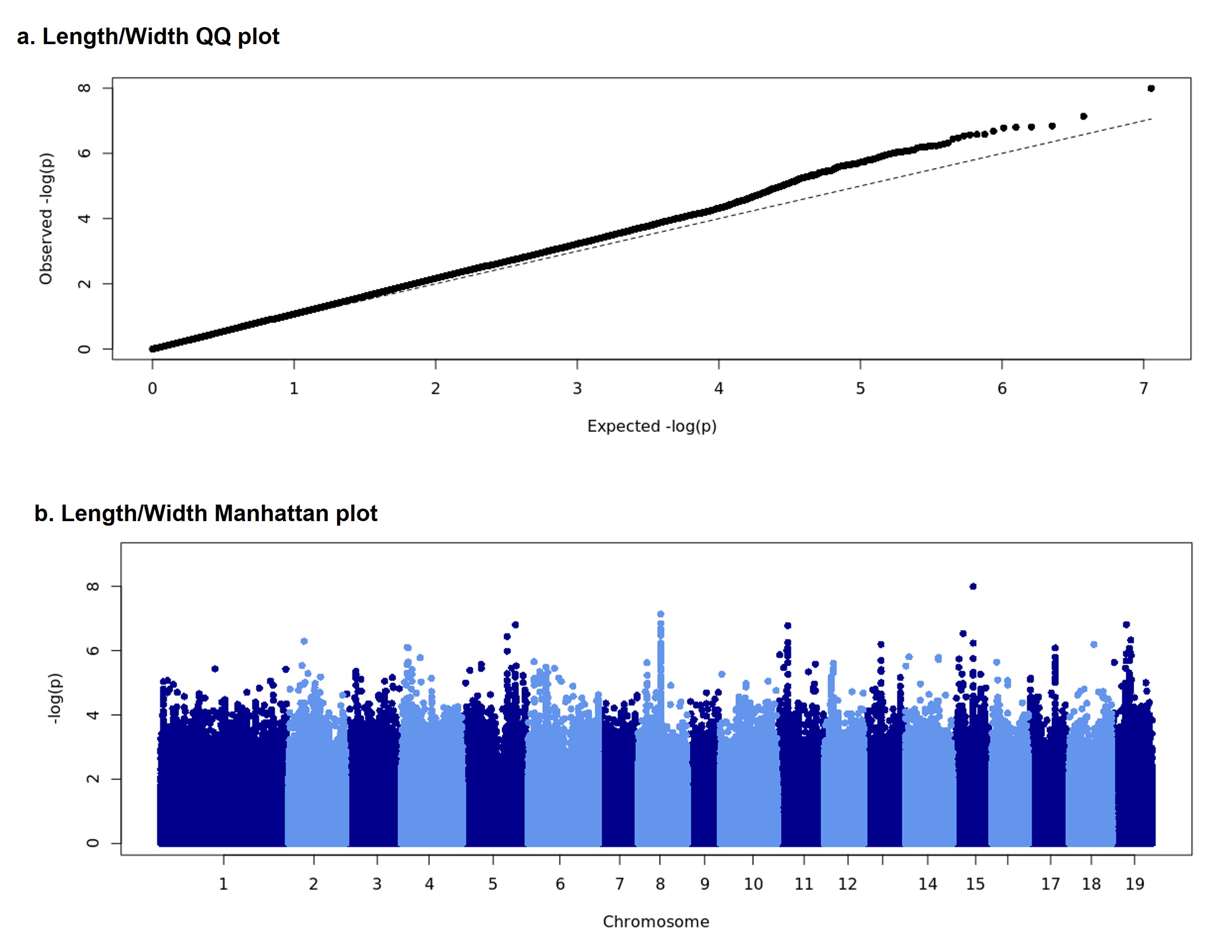

Supplement: Supplementary Figure 4 — Representative GWAS results using GEMMA linear mixed model. GWA was conducted on the leaf length/width trait using the GEMMA univariate mixed linear model. Kinship matrix was obtained using the method of VanRaden and a PCA covariate (PC1) associated with longitude (East-to-West population stratification) was included to control for cryptic population structure. Top – QQ plot. Bottom – Manhattan plot. [file Image4.jpg]

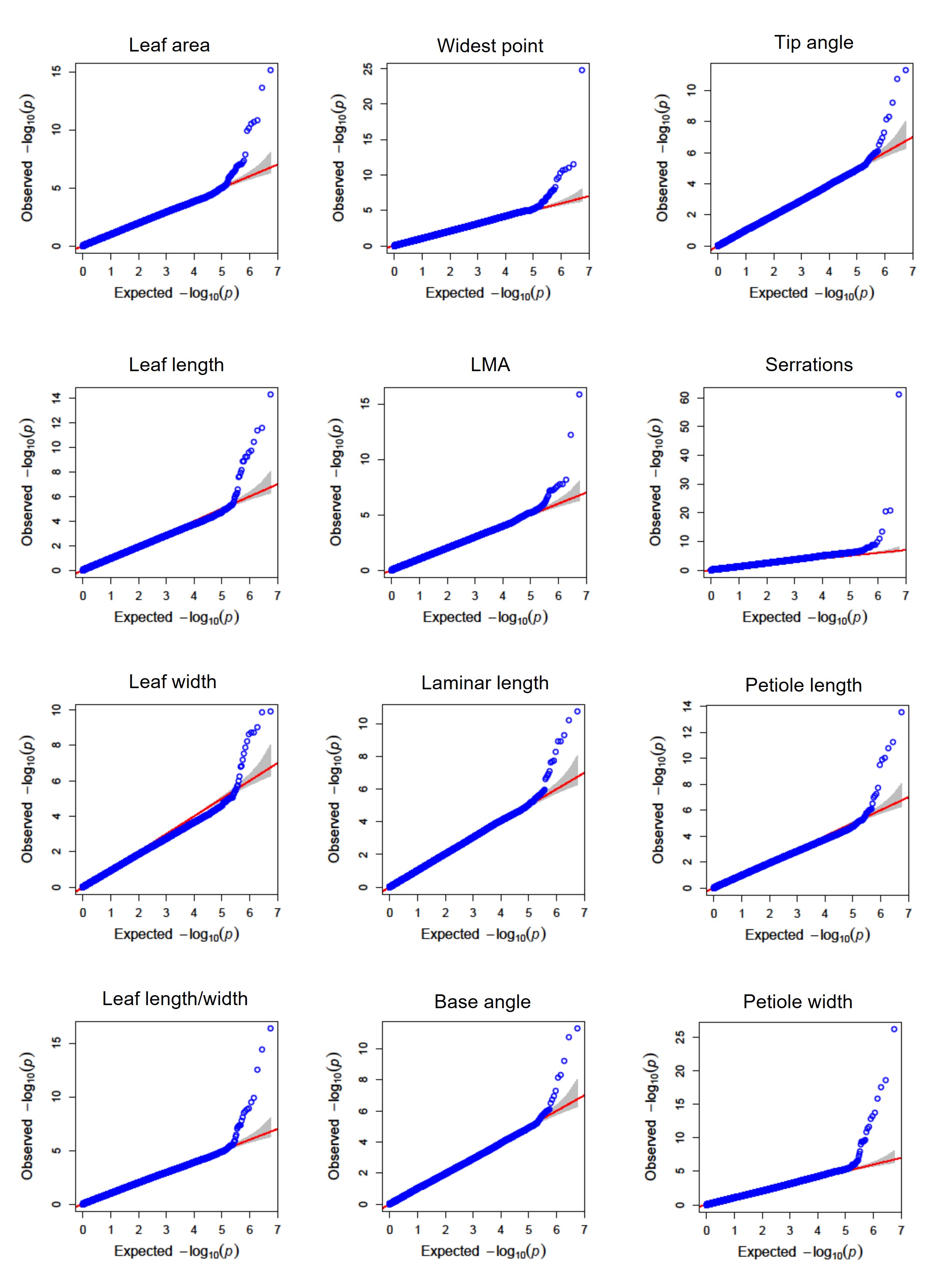

Supplement: Supplementary Figure 5 — QQ-plots illustrating fit of GWAS models conducted by FarmCPU. [file Image5.jpg]

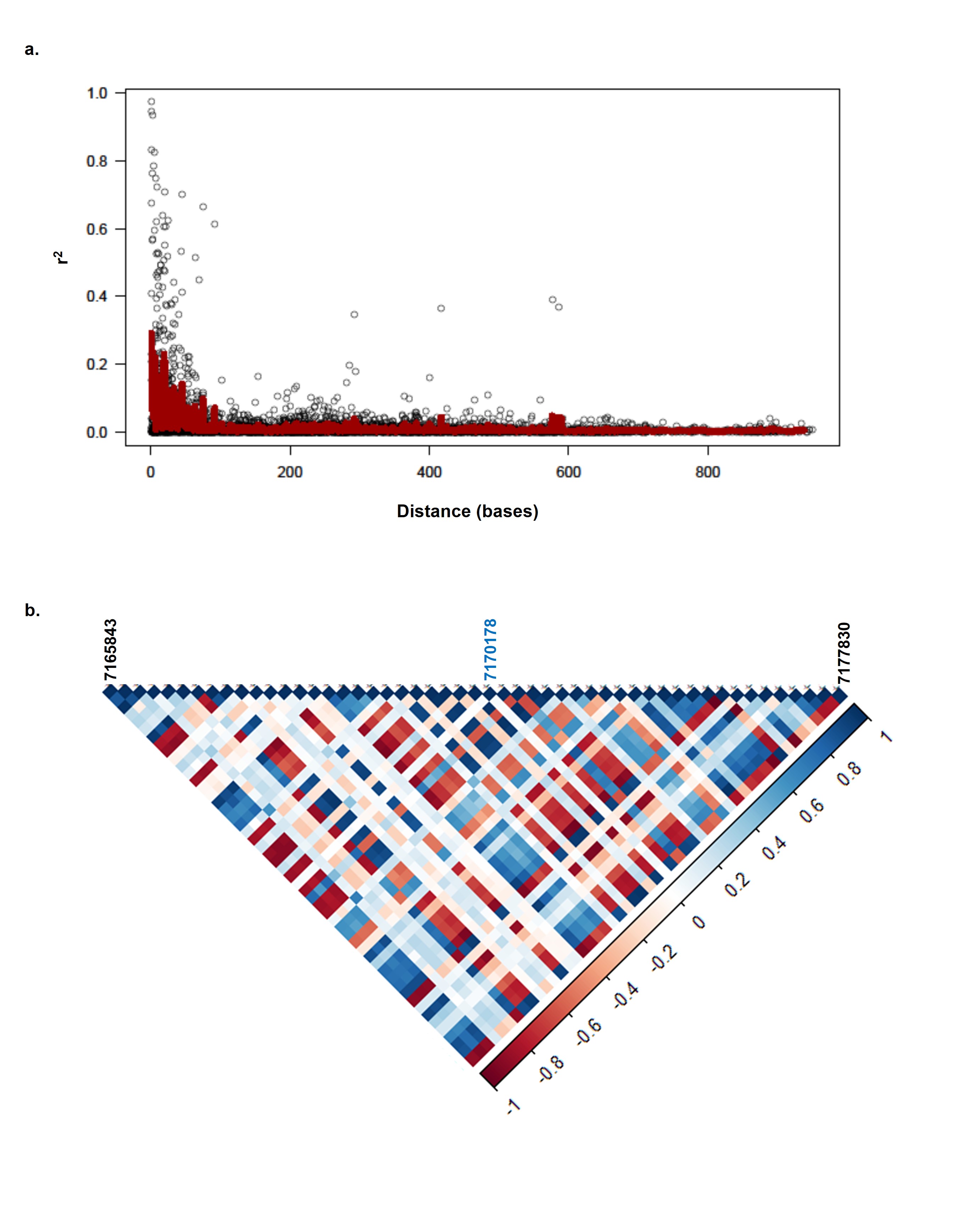

Supplement: Supplementary Figure 6 — Linkage disequilibrium in P. balsamifera. (a) Linkage disequilibrium decay plot illustrating pairwise correlation between SNPs as a function of genomic distance. (b) Linkage disequilibrium plot illustrating correlations among SNPs detected in PbTPS5. Correlations were calculated as r2. Blue colour indicates the SNP identified by GWAS. [file Image6.jpg]
